# Effect of alfalfa varieties with different resistance to alfalfa Verticillium wilt on microbial communities in rhizosphere soil and plants

**DOI:** 10.3389/fmicb.2026.1739219

**Published:** 2026-05-19

**Authors:** Li-Li Zhang, Yan-Zhong Li

**Affiliations:** State Key Laboratory of Herbage Improvement and Grassland Agro-Ecosystems, College of Pastoral Agriculture Science and Technology, Lanzhou University, Engineering Research Center of Grassland Industry, Ministry of Education, Lanzhou, China

**Keywords:** alfalfa varieties, microbial community composition, plant, rhizosphere soil, *Verticillium alfalfae*

## Abstract

Alfalfa Verticillium wilt, caused by *Verticillium alfalfae*, is a destructive disease spreading through the Northwest and central Gansu province, severely impacting alfalfa production. This study compared the community composition of rhizosphere soils from alfalfa varieties with different resistance using high-throughput sequencing. The relative abundances of *Devosia* and *Novosphingobium* were higher in the rhizosphere of the highly resistant Gannong No.4 than in Saranac. Redundancy analysis revealed that the abundances of these two bacterial genera were positively correlated with soil total nitrogen, available potassium, organic matter, and pH. Alpha diversity analysis further revealed that the rhizosphere soil of Gannong No.4 had a higher Simpson diversity index. LefSe analysis indicated that Bacillota, Bacillales, Bacillaceae, and Actinobacteria were key bacterial taxa in the rhizospheres of varieties with differing resistance. A total of 50 bacterial and 6 fungal strains were isolated from the stems and rhizosphere soils of different alfalfa varieties. Molecular characterization revealed that the bacteria comprised 48 species from 18 genera, of which 60% belonged to Bacillota and 26% belonged to Actinobacteria. Fifty bacterial strains were tested for antagonistic activity against *V. alfalfae* LYZ0257. Among these strains, seven exhibited an inhibition rate greater than 60%, and five (*Streptomyces galilaeus* LYZ1124, *Bacillus amyloliquefaciens* LYZ1125, *Bacillus velezensis* LYZ1126, *Bacillus atrophaeus* LYZ1127, and *Bacillus subtilis* LYZ1128) were as effective as the control strain (*B. amyloliquefaciens* LYZ69). Additionally, *B. amyloliquefaciens* LYZ1125, B*. velezensis* LYZ1126, *B. atrophaeus* LYZ1127, and *B. subtilis* LYZ1128 demonstrated notable capabilities in potassium solubilization, protein dissolution, and nitrogen fixation. Consequently, the rhizosphere soil of highly resistant alfalfa varieties may recruit abundant beneficial microbial communities against *V. alfalfae*.

## Introduction

Verticillium wilt is a class A quarantined disease in China (Xu et al., 2019). The pathogen is *V. alfalfae* (Inderbitzin et al., 2011) and is mainly transmitted through the soil (Li, 2021). Since it was first reported in Minle County, Zhangye City, Gansu Province, in 2014, the disease has spread further from northwestern to central Gansu province (Xu et al., 2016; Zhang and Li, 2024). When severe, it can cause a reduction of approximately 40% in both dry matter yield per plant and crude protein content in alfalfa (Li, 2021). In China, the primary approach to managing quarantine diseases is based on the principle of “prevention-oriented, integrated management” (Li and Yang, 2024). After the disease occurs, the most effective control method is to remove infected plants and fumigate the soil in affected fields with methyl bromide and chloropicrin (Xue, 2008). However, this control method not only pollutes the environment but also proves difficult to implement when the affected area is extensive (Liu, 2022). Furthermore, once pathogens enter the soil, they can persist for extended periods, making complete eradication challenging (Suo et al., 2019). Another method is breeding disease-resistant varieties. Although our team has determined that the Gannong No.4 and WL343HQ varieties have high resistance to alfalfa Verticillium wilt through greenhouse and field (Li, 2021; Yang, 2023) studies, the lengthy process of traditional breeding has yet to yield any new immune or highly resistant varieties. Therefore, the control of alfalfa Verticillium wilt remains a significant challenge.

Recently, increasing evidence has suggested that rhizosphere microorganisms play critical roles in promoting plant growth and maintaining plant health, providing the first line of defense for plants against soil-borne pathogens (Mendes et al., 2017; Levy et al., 2018; Liu et al., 2020). Previous studies have confirmed that the genotype of the host plant is one of the key factors determining the structure of the rhizosphere microbial community (Bulgarelli et al., 2013). It has also been demonstrated that the diversity of the rhizosphere microbiome exhibits heritable traits, with its community composition significantly regulated by host plant genes (Peiffer et al., 2013). Research shows that under biotic or abiotic stress, plants alter root exudates as a “cry for help” to recruit beneficial microbes with specific protective functions, thereby actively shaping their rhizosphere microbiome (Rolfe et al., 2019). This recruitment is not random, but rather a bidirectional selection process in which the host plant screens microbes via its immune and metabolic systems, and microbes adapt to compete for favorable niches (Zhang et al., 2025). Furthermore, specific microbial communities can directly enhance plants’ resistance to pathogens. There is a higher relative abundance of beneficial microorganisms in the rhizosphere soils of olives with greater resistance to disease (Fernández-González et al., 2020). The rhizosphere microbial community of a disease-resistant tomato variety was transplanted into the rhizosphere of a susceptible variety, which subsequently conferred resistance to bacterial wilt (*Ralstonia solanacearum*) in the susceptible plants (Kwak et al., 2018). This further indicated that specific rhizobacterial network structures can effectively suppress pathogen invasion (Wei et al., 2015). The study by Carrión et al. (2019) revealed that pathogen invasion can activate beneficial microorganisms with biocontrol functions within the plant root system, suggesting that a healthy beneficial microbiome has the potential to be mobilized by pathogens to counteract attacks. Liu et al. (2021) also support this view, demonstrating that disease-resistant varieties are more effective at recruiting beneficial microbes to defend against pathogens. Through high-throughput sequencing analysis of the rhizosphere soil of alfalfa plants affected by Verticillium wilt and healthy plants, our research team further revealed significant differences in the bacterial community structures of the rhizosphere soil between the two groups (Zhang and Li, 2024). However, it remains unclear whether there are differences in the microbial community composition and diversity in the rhizosphere soil of alfalfa varieties with varying resistance to alfalfa Verticillium wilt disease.

Accordingly, this study selected healthy plants from six alfalfa varieties with differing resistance to Verticillium wilt disease, cultivated in 2021 at Liuxin Village, Minle County, Zhangye City, Gansu Province (Yang, 2023). Among them, Gannong No.4 (GN) and WL343HQ (WL) are highly resistant varieties, while Dryland (HD) and Magnum II (JN) are moderately resistant varieties. Xinmu No.1 (XM) and Saranac (SA) are susceptible varieties (Yang, 2023). This study aims to perform high-throughput sequencing of the rhizosphere soil of healthy plants from different alfalfa varieties and to isolate and identify culturable microorganisms from both the rhizosphere soil and the plants. Furthermore, all isolated bacteria will be tested for their nitrogen fixation, potassium solubilization, protein degradation, and phosphate solubilization capabilities. Additionally, using *V. alfalfae* LYZ0257 as the indicator pathogen, the antagonistic effects of different bacteria against it will be evaluated. Through these experiments, the study aims to analyze the composition and diversity of rhizosphere and plant-associated microorganisms in alfalfa varieties with varying disease resistance and to provide potential biocontrol microbial resources for the biological control of alfalfa Verticillium wilt.

## Materials and methods

### Plant and soil sampling

Sampling locations were at Liu Xin Village, Minle County, Zhangye City, Gansu Province (Latitude N38°29′12.63″, Longitude E100°51′56.00, elevation 2100 m). The alfalfa plants used in this study were sown in 2021. Given that the pathogen was naturally inoculated via field infection, Verticillium wilt-infected plants were first identified in the experimental field in May 2022 (Yang, 2023). Sampling for this study was subsequently conducted in May 2023. Fifteen plants were randomly selected from GN, WL, HD, JN, XM, and SA alfalfa varieties, followed by random clipping of 5 branches from each plant. Three plants formed 1 replicate, totaling 5 replicates per variety. Soil samples were collected at a depth of 20 cm from the main root, with samples taken from the soil within 2 mm of the root surface. Fresh soil samples (0.2 g) were transferred into 2 mL sterile centrifuge tubes and frozen in liquid nitrogen. Four soil samples were selected for 16S amplification of soil bacteria at BGI Genomics, and each of the replicate samples (*n* = 4 per variety) was sequenced individually, not pooled; five samples (10 g) were used to isolate culturable bacteria, while another four samples (150 g) were used for the determination of soil properties.

### Determination of soil physicochemical properties

Soil physicochemical properties were determined using standard analytical methods. Total nitrogen content was measured using an elemental analyzer (Elementar EL Ш coupled with a Mettler AB235-S 0.0001 g precision balance). Total phosphorus, total potassium, and available potassium content were analyzed using inductively coupled plasma emission spectroscopy (ICP-OES) (Agilent 5,800 ICP-OES coupled with a Mettler ML204 0.0001 g balance). Available phosphorus content was determined using the molybdenum–antimony colorimetric method (Thermo Fisher Multiskan GO 1510 full-wavelength microplate reader coupled with a Mettler ML204 0.0001 g balance). Alkali-hydrolyzable nitrogen was analyzed via the alkali diffusion method (Prand digital bottle-neck titrator coupled with a Mettler ML204 0.0001 g balance). Organic matter content was determined using the potassium dichromate volumetric method (Prand digital bottle-neck titrator paired with a Mettler ML204 0.0001 g precision balance). Soil pH was measured using a pH meter (Leizhi PHB-4 portable pH meter paired with a Mettler ML204 0.0001 g precision balance). All determinations included four replicates, with quality control employing certified reference materials.

### Isolation of soil and plant culturable bacteria

Plant stem isolation was done as described by Zhang (Zhang and Li, 2024). For each dish, 15 branches of the tissue block were used, with 5 dishes per alfalfa variety. The nutrient agar (NA) media were placed in a 28 °C incubator in the dark and left to grow for 5 days, and the potato dextrose agar (PDA) media were placed in a 25 °C incubator. Afterward, the isolation rate statistics and culture purification were performed.

A fresh soil sample (10 g) was placed into a 150 mL conical flask containing 90 mL of sterile water. The flask was shaken (200 rpm min^−1^) for 10–15 min to evenly dissolve the soil particles. Then, 1 mL of the solution was transferred into a test tube containing 9 mL of sterile water (15 mm × 180 mm) and shaken on an SK-1 rapid mixer for 30 s to homogenize the diluent. The diluent was subsequently diluted to 10^−2^, 10^−3^, 10^−4^, and 10^−5^. The diluents of each soil sample were cultured in three plates containing NA medium for bacterial growth. The incubation temperature for bacterial growth was 28 °C. The colony number was counted after about one week, once no new colonies were observed, to determine the optimal bacterial culture gradient. A colony range of 30–200 per dish was considered ideal for further analysis. The bacteria were purified through a series of plate transfers until only a single colony appeared. The diluents of each soil sample were also cultured in three plates containing Bangladesh red medium (manufactured by Guangdong Huankai Microbial Technology Co., Ltd.) for fungal growth. The incubation temperature for fungal growth was 25 °C. After 7 days, fungal colonies in each plate were counted.Calculation of bacterial and fungal isolation rates:


$$\begin{array}{l} \text{Isolation rates}\;(\%)=\frac{\begin{array}{l} \text{The number of colonies}\\ \;\text{of}\;a\;\text{specific strain} \end{array}}{\begin{array}{l} \text{The total number}\;\\ \text{of colonies counted} \end{array}}\times 100. \end{array}$$

### DNA sequencing and phylogenetic analysis

The total genomic DNA of bacterial strains was extracted from 5-day-old cultures grown on NA using the bacteria DNA kit (B518255-0050; Shanghai Biotechnology Co., LTD) following the manufacturer’s instructions. All bacterial strains were amplified using the 27F/1429R primer pair. Polymerase chain reaction (PCR) amplification was performed on a 2,720 thermal cycler (Applied Biosystems) in a total reaction volume of 50 μL. The PCR conditions for 27F/1429R included an initial denaturation at 95 °C for 4 min, followed by 35 cycles of denaturation, primer annealing, and extension at 94 °C for 30 s, 55 °C for 30 s, and 72 °C for 1 min, respectively, with a final extension at 72 °C for 10 min (Liu and Yao, 2022).

Total genomic DNA from the fungal strains was extracted from fresh mycelia scraped from a 7-day-old culture grown on PDA using the fungal DNA kit (D3195-01; OMEGA Biotech Co. Ltd). All extraction steps followed the manufacturer’s protocol. Polymerase chain reaction (PCR) amplification was performed on a 2,720 thermal cycler (Applied Biosystems) with a total reaction volume of 25 μL using ITS1/ITS4 (White et al., 1990). The PCR conditions for ITS were as follows: an initial denaturation at 95 °C for 3 min, followed by 35 cycles at 95 °C for 30 s, 55 °C for 30 s, and 72 °C for 1 min, with a final extension at 72 °C for 7 min (White et al., 1990).

The PCR products were loaded into 1% agarose gels for electrophoresis, and the amplified products were sent to Sangon Biotech (Shanghai, China) for purification and sequencing. Sequencing data were assembled with DNAMAN v. 5.2.2 (LynnoBiosoft). Sequences were submitted to GenBank, and accession numbers were obtained (Cao et al., 2020).

### Genomic DNA extraction and sequencing

Genomic DNA from GN, WL, JN, HD, XM, and SA soil was extracted using either the cetyltrimethylammonium bromide (CTAB) or sodium dodecyl sulfate (SDS) method. The purity and concentration of the extracted DNA were assessed using agarose gel electrophoresis. The DNA samples were then normalized to a concentration of 1 ng·μL^−1^ using sterile water and used as templates for PCR. PCR was performed using phusion® high-fidelity PCR master mix with GC buffer and high- efficiency, high-fidelity enzymes (New England Biolabs) to ensure amplification accuracy. Specific primers with barcodes were synthesized to amplify the bacterial 16S rRNA V4-V5 region (515F: 5′-GTGCCAGCMGCCGCGGTAA-3′ and 806R: 5′-GGACTACHVGGGTWTCTAAT-3′). Each PCR reaction consisted of 15 μL of phusion® high-fidelity PCR master mix (New England Biolabs), 0.2 μM of both forward and reverse primers, and approximately 10 ng of template DNA. The thermal cycling conditions included an initial denaturation at 98 °C for 1 min, followed by 30 cycles of denaturation at 98 °C for 10 s, primer annealing at 50 °C for 30 s, and elongation at 72 °C for 30 s, with a final extension at 72 °C for 5 min. The PCR products were mixed with 1X loading buffer (containing SYBR green) and analyzed on a 2% agarose gel to check for the presence of the target bands. The PCR products were subsequently combined in equal density ratios and purified using the Universal DNA Purification Kit (TianGen, China). Sequencing libraries were prepared using the NEBNext® Ultra™ II FS DNA PCR-free library prep kit (New England Biolabs, USA), following the manufacturer’s instructions. The library was quantified using a Qubit fluorometer and real-time PCR, and its size distribution was assessed using a bioanalyzer. Quantified libraries were pooled and sequenced on an Illumina NovaSeq6000 platform according to the required library concentration and data amount.

### Bioinformatics analysis

The paired-end reads were assigned to samples based on their unique barcodes. The barcodes and primer sequences were then trimmed, and the reads subsequently merged using FLASH (Magoč and Salzberg, 2011). FLASH was used because it is fast and accurate for merging paired-end reads when overlapping sequences are present between reads from opposite ends of the same DNA fragment. The merged sequences were referred to as raw tags and were subsequently filtered for quality using fastp (version 0.23.1) to generate high-quality clean tags (Bokulich et al., 2012). The clean tags were then compared to reference databases (silva database for 16S/18S, unite database for ITS) using the UCHIME algorithm to detect and remove chimera sequences (Edgar et al., 2011), yielding effective tags. Denoising of the effective tags was performed using the DADA2 module in the QIIME2 software (version QIIME2-202202) to generate initial ASVs (amplicon sequence variants). Species annotation was conducted using QIIME2 software. 16S / 18S annotations were done using the silva database. The micro-NT database, which is a sub-library containing bacteria extracted from the NT database, was used by default for irregular regions. The absolute abundance of ASVs was normalized to match the sequence number of the sample with the fewest sequences. Subsequent analyses, including alpha diversity and beta diversity, were based on the normalized data. The top 10 taxa at the genus level for each sample were selected, and a distribution histogram of their relative abundance was plotted using Origin 2018 with the SVG function.

### Nitrogen fixation capacity determination

The nitrogen-free medium (HB8752, produced by Haibo Biotechnology Co., Ltd., Qingdao Hi-Tech Industrial Park) was used to evaluate the nitrogen-fixing potential of all bacterial strains via a serial transfer method. Strains activated in Lysogeny Broth (LB) solid medium were inoculated into the nitrogen-free medium (in triplicate) and cultured at 28 °C for 7 days. This process was repeated three times consecutively. If colonies formed in the medium after each passage, the strain was determined to possess nitrogen-fixing ability (Li L. L. et al., 2023).

### Determination of phosphorus solubilization capacity

All test bacteria were activated on LB medium and then individually inoculated by spot plating onto inorganic phosphorus medium (HB8549-2, produced by Haibo Biotechnology Co., Ltd., Qingdao Hi-Tech Industrial Park) and organic phosphorus medium (HB8549-1, produced by Haibo Biotechnology Co., Ltd., Qingdao Hi-Tech Industrial Park) (three replicates per treatment). Cultures were incubated at 28 °C for 10 days. Phosphate solubilization capacity was qualitatively assessed by observing the presence of solubilization zones (Li L. L. et al., 2023). For quantitative evaluation, after 14 days of incubation, the diameter of the solubilization zone (D) and the diameter of the bacterial colony (d) were measured. The D/d ratio was calculated to preliminarily evaluate the phosphate solubilization capacity of each strain.

### Functional characterization of proteases

Protease activity of test bacteria was evaluated using the skim milk agar plate method (Xu, 2023). All strains were inoculated at a spot count onto plates (skim milk agar medium, produced by Qingdao High-Tech Industrial Park Haibo Biotechnology Co., Ltd.). After incubation at 37 °C for 1–2 days, the formation of hydrolysis zones (clear zones) was observed. The diameter of the hydrolysis zone (D) and the diameter of the bacterial colony (d) were measured. The proteolytic activity of each strain was determined by calculating the D/d ratio (HC).

### Potassium release function identification

The potassium-solubilizing ability of test bacteria was evaluated using the plate method with silicate bacterial medium (HB8548-1, produced by Qingdao High-Tech Industrial Park Haibo Biotechnology Co., Ltd.) (Xu, 2023). After triple-streak inoculation, cultures were incubated at 37 °C for 3 to 5 days. If colonies exhibited a smooth, transparent, droplet-like morphology with a capsule, the strain was confirmed to possess potassium-solubilizing ability.

### Bacteriostatic activity assay for culturable bacteria

The inhibition of fifty-one bacterial strains against *V. alfalfae* LYZ0257 was determined. We chose *Bacillus amyloliquefaciens* LYZ69 as the control strain due to its superior biocontrol effect against alfalfa anthracnose (*Colletotrichum truncatum*) (Hu et al., 2021). A mycelial plug of pathogenic fungi (diameter = 5 mm) was placed at the center of the PDA plate, and the antagonist strain to be tested was applied to four points around the plug to form a “+” shape. Each point was 2 cm away from the plug. The plate inoculated with the pathogenic fungus alone, without the antagonist, was used as the control. The diameter of the inhibition ring and the growth of the colony were measured using a vernier caliper after the control colony had fully covered the plate. The experiment was conducted in triplicate, and the growth inhibition rate of the colonies was calculated using Equation 1:


$$\begin{array}{l} \text{Colony growth inhibition rate}\%=\\ \frac{\left(\begin{array}{l} \text{diameter of control colony}-\\ \text{diameter of treated colony} \end{array}\right)}{\text{diameter of control colony}}\times 100\% \end{array}$$ (1)

### Data analysis

Alpha diversity was calculated using four indexes in QIIME2: Chao1, Shannon, Simpson, and ACE, to analyze the diversity, richness, and evenness of the communities in the samples.

Beta diversity was calculated based on unweighted UniFrac distances in QIIME2 to evaluate the complexity of community composition and compare differences between samples (or groups). This analysis was conducted using principal component analysis (PCA) to reduce the dimensionality of the original variables. It was performed using the ade4 and ggplot2 packages in R software (version 4.0.3). PERMANOVA and ANOSIM were subsequently used to assess the Bray–Curtis distances of the ASVs of different treatments using the “vegan” package in R. When performing multiple pairwise comparisons (PERMANOVA and ANOSIM), we applied the False Discovery Rate (FDR) correction method (Benjamini–Hochberg procedure) to adjust the *p*-values, thereby controlling the false positive risk associated with multiple comparisons.

Redundancy analysis (RDA) was conducted using Canoco 5 software to explore the primary physicochemical drivers affecting bacterial structure.

### Availability of data and materials

All clean sequencing data from this project on rhizosphere soil are available in the NCBI Sequence Read Archive (SRA) database under bioproject PRJNA1223621, with detailed information provided in Supplementary Tables S1, S2. Culturable bacteria from rhizosphere soil 16S sequencing data are available under bioprojects PV110947 to PV110975, and culturable bacteria from alfalfa stems 16S sequencing data are available under bioprojects PV110989 to PV111008.

## Results

### Bacterial and fungi composition in soil

**Figure 1:**
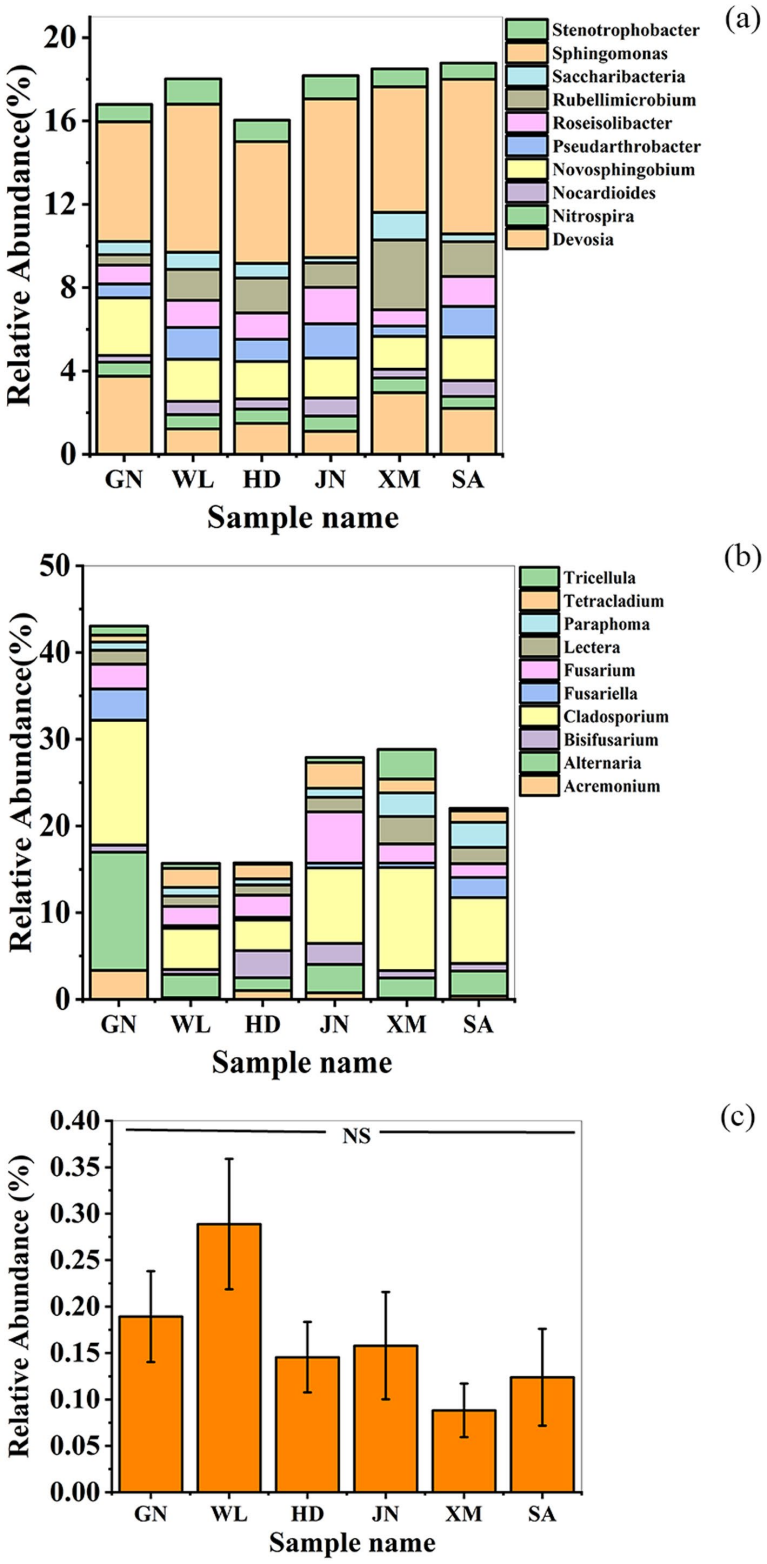
The relative abundance of the top 10 genera of soil bacteria (**a**) and fungi (**b**). Relative abundance of *Bacillus* among different varieties (**c**). GN: Gannong No.4 alfalfa variety; WL: WL343HQ alfalfa variety; HD: Dryland alfalfa variety; JN: Magnum II; XM: Xinmu No.1 alfalfa variety; SA: Saranac alfalfa variety.

The top 10 bacterial genera by relative abundance in the rhizosphere soil of the six alfalfa varieties were *Devosia*, *Nitrospira*, *Nocardioides*, *Novosphingobium*, *Pseudarthrobacter*, *Roseisolibacter*, *Rubellimicrobium*, *Saccharibacteria*, *Sphingomonas*, and *Stenotrophobacter*. Among these, the relative abundances of *Devosia* (3.75% ± 1.75%) and *Novosphingobium* were higher in the resistant variety GN than in the susceptible varieties XM and SA. WL exhibited the highest abundance of *Stenotrophobacter* among all varieties. *Rubellimicrobium* was significantly enriched in XM, whereas SA showed elevated levels of *Pseudarthrobacter* and *Sphingomonas* compared to the other varieties (Figure 1A, Table 1).

**Table 1:** Top 10 bacterial genera in relative abundance in rhizosphere soil.

Genes	Gannong No.4	WL343HQ	Dryland	Magnum II	Xinmu No.1	Saranac
*Devosia*	3.75 ± 1.75a	1.22 ± 0.34a	1.48 ± 0.37a	1.10 ± 0.23a	2.96 ± 1.48a	2.20 ± 1.29a
*Nitrospira*	0.67 ± 0.19a	0.69 ± 0.06a	0.69 ± 0.13a	0.74 ± 0.11a	0.70 ± 0.11a	0.57 ± 0.09a
*Nocardioides*	0.32 ± 0.08c	0.63 ± 0.12abc	0.49 ± 0.13abc	0.87 ± 0.22a	0.42 ± 0.08bc	0.76 ± 0.04ab
*Novosphingobium*	2.77 ± 0.64a	2.02 ± 0.28a	1.79 ± 0.54a	1.91 ± 0.64a	1.58 ± 0.15a	2.09 ± 0.29a
*Pseudarthrobacter*	0.65 ± 0.08b	1.53 ± 0.24a	1.07 ± 0.18ab	1.65 ± 0.26a	0.50 ± 0.08b	1.46 ± 0.24a
*Roseisolibacter*	0.92 ± 0.36ab	1.30 ± 0.22ab	1.26 ± 0.07ab	1.74 ± 0.31a	0.77 ± 0.31b	1.44 ± 0.30ab
*Rubellimicrobium*	0.50 ± 0.29a	1.49 ± 0.31a	1.67 ± 1.30a	1.18 ± 0.57a	3.36 ± 2.09a	1.67 ± 0.54a
*Saccharibacteria*	0.62 ± 0.20a	0.82 ± 0.44a	0.71 ± 0.41a	0.26 ± 0.13a	1.32 ± 1.12a	0.37 ± 0.17a
*Sphingomonas*	5.75 ± 1.21a	7.11 ± 0.94a	5.85 ± 1.73a	7.61 ± 2.09a	6.02 ± 1.72a	7.43 ± 1.04a
*Stenotrophobacter*	0.84 ± 0.28a	1.21 ± 0.17a	1.02 ± 0.22a	1.11 ± 0.21a	0.87 ± 0.27a	0.78 ± 0.12a

Different letters within the same line indicate significant differences in relative abundance of rhizosphere soil among different varieties (*p* < 0.05).

The top 10 fungal genera by relative abundance in rhizosphere soil were *Acremonium*, *Alternaria*, *Fusarium*, *Cladosporium*, F*usariella*, *Lectera*, *Paraphoma*, *Tetracladium*, and *Tricellula*. In the highly resistant variety GN, the relative abundances of *Acremonium*, *Alternaria*, and *Cladosporium* were higher than those in XM and SA (Figure 1B, Table 2).

**Table 2:** Top 10 fungi genera in relative abundance in rhizosphere soil.

Genes	Gannong No.4	WL343HQ	Dryland	Magnum II	Xinmu No.1	Saranac
*Acremonium*	3.35 ± 2.26a	0.21 ± 0.08a	1.03 ± 0.62a	0.77 ± 0.54a	0.16 ± 0.05a	0.39 ± 0.25a
*Alternaria*	13.63 ± 7.95a	2.69 ± 0.95b	1.48 ± 0.96b	3.26 ± 1.81b	2.32 ± 0.81b	2.89 ± 0.87b
*Bisifusarium*	0.83 ± 0.27a	0.55 ± 0.18a	3.11 ± 2.88a	2.44 ± 0.38a	0.83 ± 0.19a	0.86 ± 0.51a
*Cladosporium*	14.38 ± 9.38a	4.74 ± 1.04a	3.55 ± 1.90a	8.70 ± 4.28a	11.90 ± 6.57a	7.60 ± 2.43a
*Fusariella*	3.62 ± 3.04a	0.30 ± 0.18a	0.29 ± 0.21a	0.55 ± 0.23a	0.51 ± 0.20a	2.33 ± 1.76a
*Fusarium*	2.85 ± 0.47b	2.24 ± 0.54b	2.56 ± 0.69b	5.92 ± 1.71a	2.21 ± 0.74a	1.58 ± 0.40a
*Lectera*	1.62 ± 0.83	1.19 ± 0.37	1.16 ± 0.42	1.68 ± 0.46	3.15 ± 1.13	1.90 ± 0.74
*Paraphoma*	0.94 ± 0.51a	1.00 ± 0.53a	0.71 ± 0.29a	1.04 ± 0.23a	2.73 ± 0.50a	2.89 ± 1.37a
*Tetracladium*	0.78 ± 0.45a	2.19 ± 0.73a	1.70 ± 0.56a	2.96 ± 1.93a	1.60 ± 0.44a	1.29 ± 0.61a
*Tricellula*	1.03 ± 0.67b	0.58 ± 0.37b	0.16 ± 0.07b	0.60 ± 0.37b	3.41 ± 1.42b	0.31 ± 0.12a

Different letters within the same line indicate significant differences in relative abundance of rhizosphere soil among different varieties (*p* < 0.05).

The relative abundance of *Bacillus* in the rhizosphere soil of different alfalfa varieties showed no significant differences. However, the highly resistant varieties WL343HQ and Gannong No.4 exhibited a higher relative abundance compared to the moderately resistant variety Juneng No.2 and the drought-tolerant varieties, as well as the susceptible varieties Xinmu No.1 and Saranac (Figure 1c).

### Evaluation of sequencing depth sufficiency

To assess whether the sequencing depth was sufficient to capture the majority of microbial diversity, cumulative curves were generated for both bacterial and fungal communities based on the number of samples sequenced. The number of detected OTUs for both bacteria and fungi increased progressively with the addition of more samples. The bacterial cumulative curve exhibited a steep initial rise, reaching approximately 4,000 OTUs after 10 samples, and gradually plateaued around 4,851 OTUs after 24 samples (Supplementary Figure S1a). Similarly, the fungal cumulative curve showed a rapid increase in OTU detection during the early sampling stages, with around 1,100 OTUs detected by the 10 samples, and approached saturation at approximately 1,299, OTUs after 24 samples (Supplementary Figure S1b). These patterns indicate that the sampling effort was adequate to capture the vast majority of bacterial and fungal OTUs in the rhizosphere soil, with only a minimal number of new OTUs expected with additional sequencing. This suggests that the sequencing depth was sufficient for downstream diversity and community structure analyses.

### Alpha diversity of bacterial communities in soil

**Figure 2:**
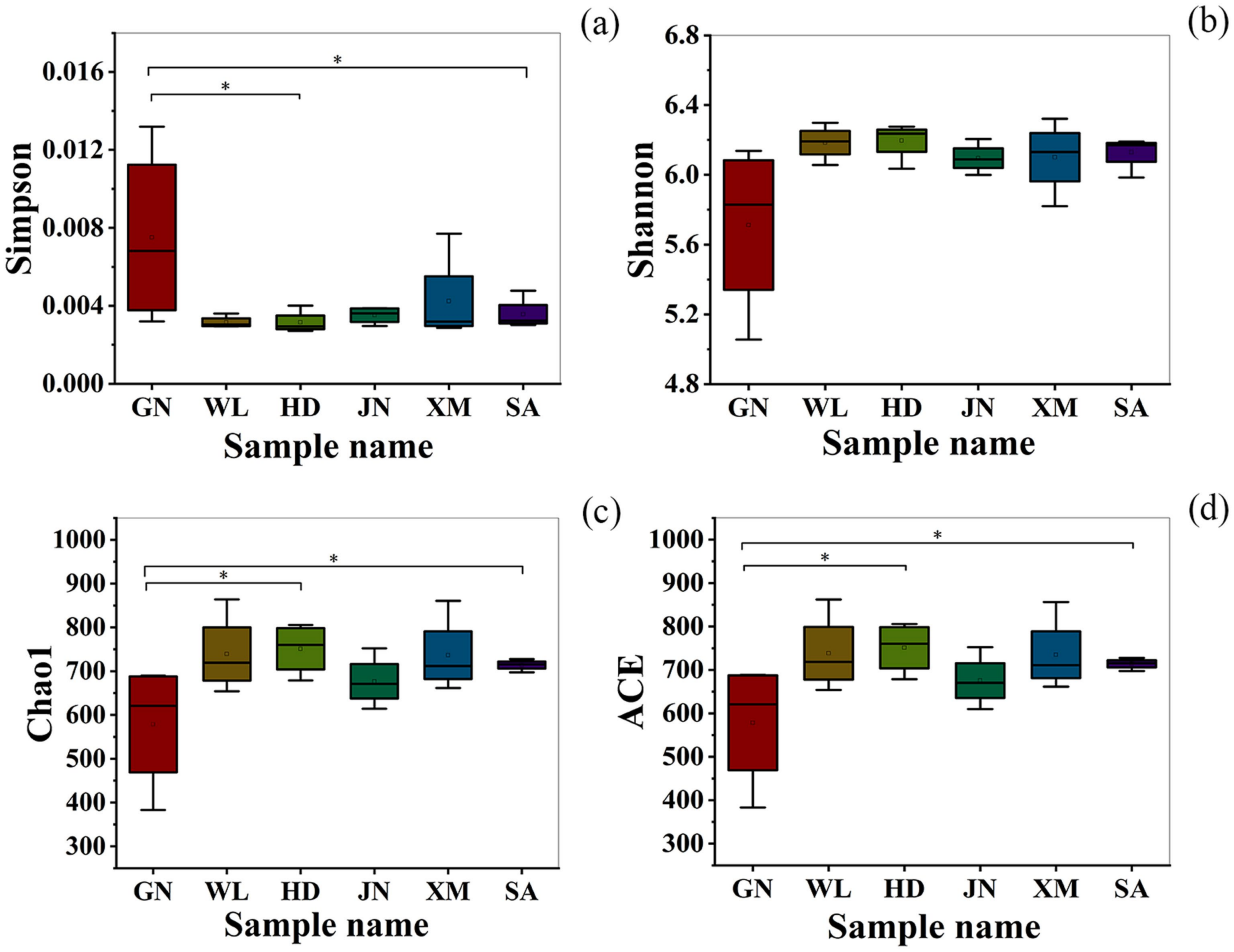
The alpha diversity index of the soil bacterial communities under different varieties. **(a)** Simpson diversity index. **(b)** Shannon diversity index. **(c)** Chao1 richness index. **(d)** ACE richness index. “*” indicates significant differences in alpha diversity indices among alfalfa varieties (*p* < 0.05). GN: Gannong No.4 alfalfa variety; WL: WL343HQ alfalfa variety; HD: Dryland alfalfa variety; JN: Magnum II; XM: Xinmu No.1 alfalfa variety; SA: Saranac alfalfa variety.

The Simpson diversity index (Figure 2a), Shannon diversity index (Figure 2b), and Chao1 richness index (Figure 2c) of rhizosphere soil bacteria among different alfalfa varieties showed no significant differences. However, the Simpson diversity index of rhizosphere soil bacteria in the GN alfalfa variety was significantly higher than that in Chao1 richness index (Figure 2c), and ACE richness index (Figure 2d) in rhizosphere soils of different alfalfa varieties showed no significant differences. However, the Simpson index of rhizosphere soil bacteria in GN alfalfa was significantly higher than that in moderately drought-tolerant and susceptible SA, while the Chao1 and ACE indices showed the opposite trend (*p* < 0.05) (Table 3).

**Table 3:** The alpha diversity index of the bacteria and fungi of rhizosphere soil.

Alpha diversity index	Bacteria		Fungi	
	*F*	*p*	*F*	*p*
Chao1	2.323	0.086	1.137	0.377
ACE	2.334	0.084	1.137	0.377
Shannon	2.413	0.077	0.682	0.643
Simpson	2.397	0.078	0.624	0.684

Simpson diversity index (Supplementary Figure S2a), Shannon diversity index (Supplementary Figure S2b), Chao1 richness index (Supplementary Figure S2c), and ACE richness index (Supplementary Figure S2d) of rhizosphere soil fungi showed no significant differences among alfalfa varieties (Table 3).

### Beta diversity of bacterial communities in soil

**Figure 3:**
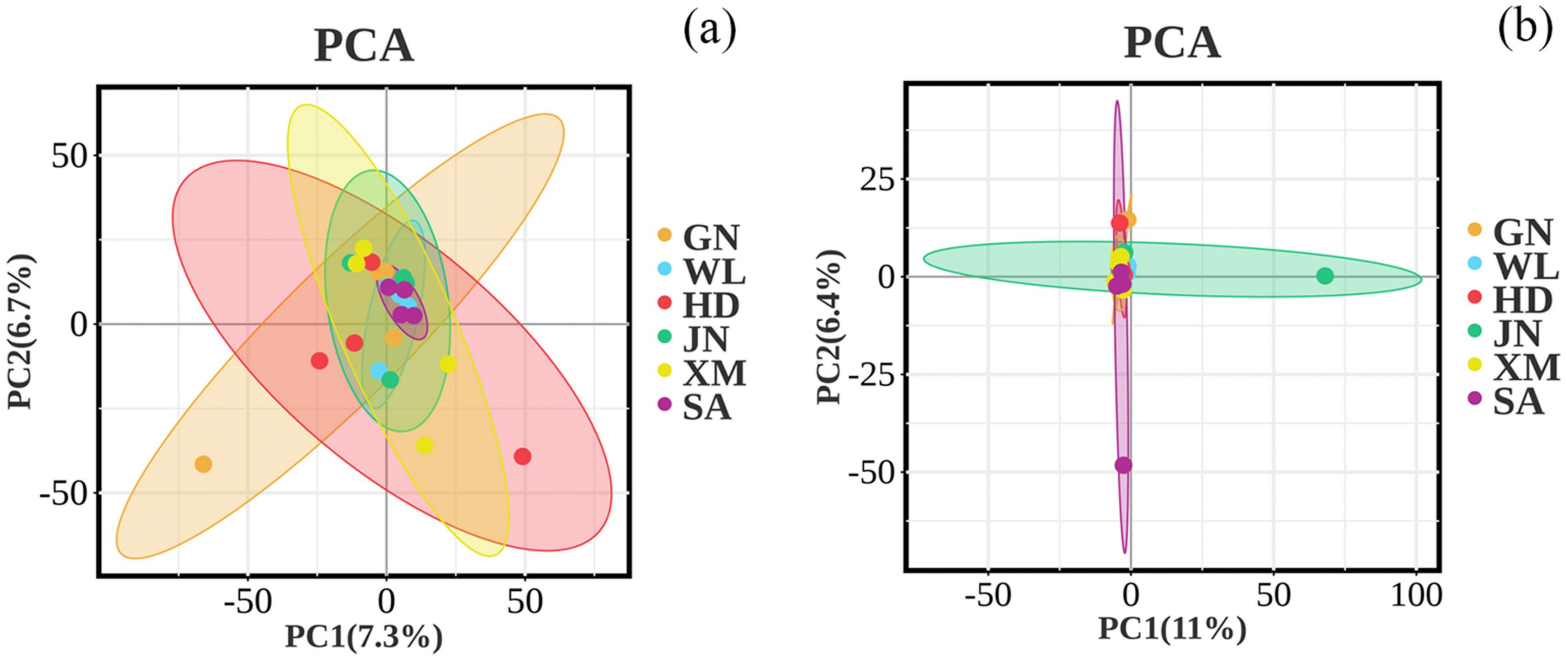
Principal component analysis (PCA) of the soil bacterial and fungal structures in different varieties. **(a)** Bacterial. **(b)** Fungi. GN: Gannong No.4 alfalfa variety; WL: WL343HQ alfalfa variety; HD: Dryland alfalfa variety; JN: Magnum II; XM: Xinmu No.1 alfalfa variety; SA: Saranac alfalfa variety.

ANOSIM and PERMANOVA analyses revealed that the composition of the bacteria (Figure 3a) and fungi (Figure 3b) among different varieties was not significantly different between GN and HD, GN and JN, GN and XM, GN and SA, WL and HD, WL and JN, WL and XM, WL and SA, HD and XM, HD and SA, JN and XM, and JN and SA (Table 4).

**Table 4:** The statistical test of analysis of similarity (ANOSIM) and permutational multivariate one-way analysis of variance (PERMANOVA) to analyze differences in rhizosphere soil bacteria and fungi community compositions measured by amplicon sequencing under different treatments.

Group	Degree of freedom	Bacteria					Fungi				
		PERMANOVA			ANOSIM		PERMANOVA			ANOSIM	
		*F*	*p*	p*	*R*	*p*	*F*	*p*	p*	*R*	*p*
Gannong No.4 vs WL343HQ vs Dryland vs Magnum II vs Xinmu No.1 vs Saranac	5	0.98	0.542		−0.015	0.597	0.92	0.616		−0.028	0.643
Gannong No.4 vs. Dryland	1	1.008	0.435	0.768	0.01	0.432	0.908	0.463	0.765	−0.104	0.664
Gannong No.4 vs Magnum II	1	0.957	0.483	0.768	−0.063	0.721	0.544	1	1	−0.24	0.943
Gannong No.4 vs. Xinmu No.1	1	0.952	0.502	0.563	0.094	0.243	0.986	0.361	0.765	0	0.422
Gannong No.4 vs. Saranac	1	0.974	0.563	0.756	0.021	0.367	0.924	0.531	0.765	0	0.409
WL343HQ vs Dryland	1	0.864	0.756	0.81	0.021	0.384	0.92	0.541	0.765	−0.021	0.529
WL343HQ vs Magnum II	1	0.83	0.811	0.811	−0.156	0.807	1.449	0.131	0.765	0.094	0.212
WL343HQ vs Xinmu No. 1	1	1.169	0.3	0.768	0.26	0.141	1.113	0.302	0.765	0.063	0.312
WL343HQ vs Saranac	1	1.066	0.346	0.768	0	0.527	0.694	0.639	0.799	−0.146	0.787
Dryland vs Xinmu No.1	1	0.952	0.315	0.768	−0.042	0.368	0.941	0.502	0.765	−0.063	0.655
Dryland vs Saranac	1	0.857	0.749	0.81	−0.156	0.803	0.794	0.561	0.765	−0.063	0.529
Magnum II vs Xinmu No.1	1	0.913	0.48	0.768	−0.021	0.435	1.059	0.343	0.765	0.052	0.323
Magnum II vs Saranac	1	0.936	0.619	0.774	−0.094	0.756	0.882	0.877	0.94	−0.042	0.505

“*” indicates that the *p*-values were adjusted using the False Discovery Rate (FDR) correction method (Benjamini–Hochberg procedure).

### LEfSe analysis of rhizosphere soil bacteria and fungi

**Figure 4:**
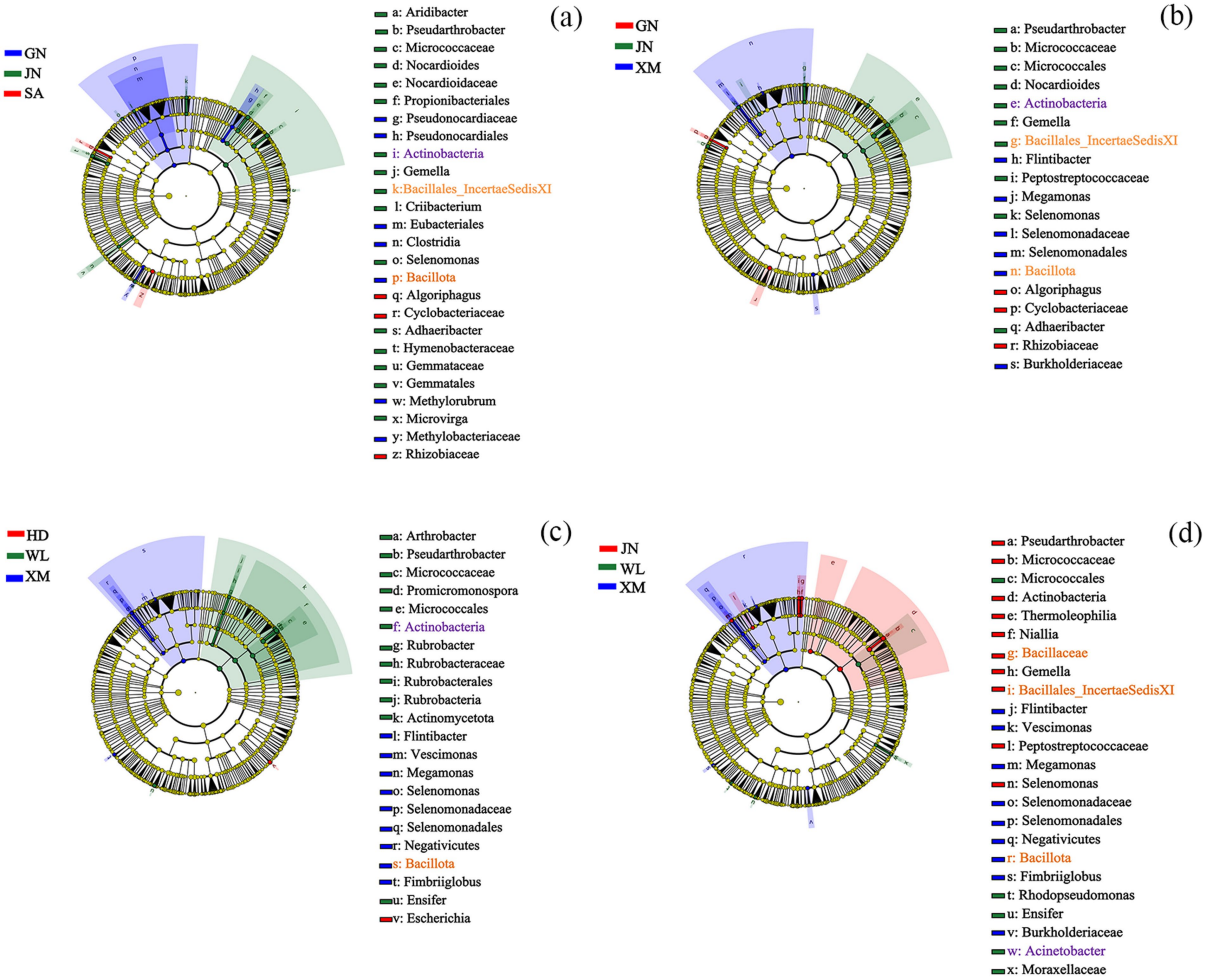
Cladogram indicating the phylogenetic distribution of microbial lineages under different alfalfa varieties, with each circle’s diameter proportional to the given taxon’s relative abundance. Phylogenetic distribution of bacterial lineages under GN, JN, and SA **(a)**; Phylogenetic distribution of bacterial lineages under GN, JN, and XM **(b)**; Phylogenetic distribution of bacterial lineages under HD, WL, and XM **(c)**; Phylogenetic distribution of bacterial lineages under WL, JN, and XM **(d)**. GN: Gannong No.4 alfalfa variety; WL: WL343HQ alfalfa variety; HD: Dryland alfalfa variety; JN: Magnum II; XM: Xinmu No.1 alfalfa variety; SA: Saranac alfalfa variety.

Key microbial groups in the rhizosphere soil differ among alfalfa varieties. The GN-JN-SA comparison harbored 26 taxonomic groups (Figure 4a), the GN-JN-XM comparison contained 19 (Figure 4b), the WL-HD-XM comparison included 22 (Figure 4c), and the WL-XM-SA comparison included 24 (Figure 4d), spanning multiple phyla, classes, orders, families, genera, and species. Additionally, the phylum Bacillota was a key microbial group for both GN and XM; the class Bacillales and family Bacillaceae were important for JN; and the phylum Actinobacteria was significant for both WL and JN.

LEfSe analysis revealed significantly different taxonomic biomarkers among the various comparison groups for rhizosphere soil bacteria. The LEfSe analysis identified distinct bacterial taxa that were significantly enriched in the rhizosphere soil among the GN-JN-SA (Supplementary Figure S3a) and GN-JN-XM (Supplementary Figure S3b) comparisons. At the phylum level, Bacillota exhibited higher relative abundances in SA compared to GN and JN. Within the class level, Actinobacteria showed consistent enrichment patterns, with the highest LDA scores observed in JN. The LEfSe analysis also identified distinct bacterial taxa that were significantly enriched in the rhizosphere soil among the WL-HD-XM (Supplementary Figure S3c) comparison. At the phylum level, Bacillota exhibited higher relative abundances in XM compared to WL and HD. Within the class level, Actinobacteria showed consistent enrichment patterns, with the highest LDA scores observed in JN (Supplementary Figure S3d).

LEfSe analysis revealed significantly different taxonomic biomarkers among the various comparison groups (rhizosphere soil fungi). In WL-JN, significant enrichment was observed at multiple taxonomic levels: The family Leptosphaeriaceae and its genus *Leptosphaeria* exhibited high LDA scores, alongside the class Orbiliomycetes, the order Orbiliales, and the family Orbiliaceae (Supplementary Figure S4a). For the JN-XM group, the genus *Paraphoma* emerged as the most discriminant biomarker, showing the highest LDA score among all taxa (Supplementary Figure S4b). In the HD-SA comparison, the order Diversisporales, along with its corresponding family Diversisporaceae and genus Diversispora, were identified as significantly enriched taxa, with the order Agaricales also serving as a key co-enriched biomarker (Supplementary Figure S4c). Lastly, in the HD-XM group, the family Phaeosphaeriaceae and the class Leotiomycetes were both identified as significantly enriched biomarkers (Supplementary Figure S4d).

### Relationship between soil microbial community structure and soil biological properties

**Figure 5:**
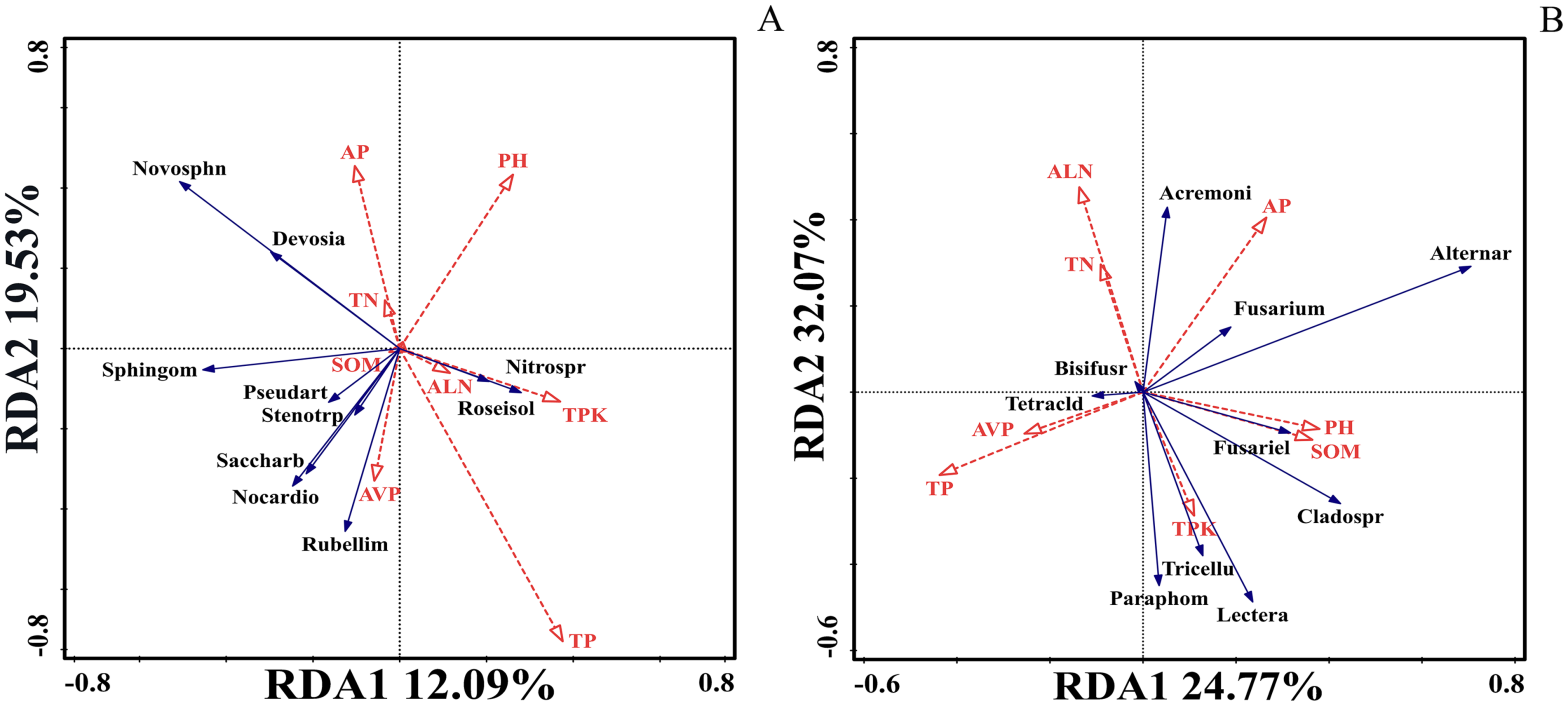
RDA of the top 10 bacteria (fungi) by relative abundance in soil microorganisms and soil physicochemical properties. **(A)** Top 10 bacterial genera by relative abundance (Novosphn: *Novosphingobium*, Nitrospr: *Nitrospira*, Roseisol: *Roseisolibacter*, Rubellim: *Rubellimicrobium*, Nocardio: *Nocardioides*, Saccharb: *Saccharibacteria*, Stenotroph: *Stenotrophobacter*, Pseudarth: *Pseudarthrobacter*, Sphingom: *Sphingomonas*) with soil total nitrogen (TN), pH, alkaline-hydrolyzable nitrogen (ALN), total potassium (TPK), total phosphorus (TP), available potassium (AP), soil organic matter (SOM), and available phosphorus (AVP) using RDA. **(B)** The top 10 fungal genera by relative abundance (Acremoni: *Acremonium*, Alternar: *Alternaria*, Fusariel: *Fusariella*, Cladospr: *Cladosporium*, Tricellu: *Tricellula*, Paraphom: *Paraphoma*, Tetracld: *Tetracladium*, Bisifusr: *Bisifusarium*) with soil total nitrogen (TN), pH, alkaline-hydrolyzable nitrogen (ALN), total potassium (TPK), total phosphorus (TP), available potassium (AP), soil organic matter (SOM), and available phosphorus (AVP) using RDA.

The high relative abundance of bacterial genera *Devosia* and *Novosphingobium* was positively correlated with soil TN, AP, SOM, and pH, but negatively correlated with ALN, TPK, AVP, and TP. *Pseudarthrobacter*, *Stenotrophobacter*, *Saccharibacteria*, *Nocardioides*, and *Rubellimicrobium* showed significant positive correlations with SOM, AVP, and TP (Figure 5A).

The fungal genera with relatively high abundance, specifically *Alternaria*, *Fusarium*, *Acremonium*, and *Fusariella*, showed significant positive correlations with AP, pH, and SOM. Meanwhile, *Tricellula*, *Paraphoma*, *Fusariella*, *Cladosporium*, and *Lectera* showed significant positive correlations with TPK, SOM, and pH (Figure 5B).

### Identification of culturable microorganisms

A total of 29 bacterial strains were isolated from the rhizosphere soil of different alfalfa varieties (Supplementary Figure S5, Table S3). Molecular identification using primers 27F/1429R identified 27 species belonging to fourteen genera. Additionally, six fungal strains were isolated and identified using primers ITS1/ITS4, revealing six species belonging to five genera (Supplementary Table S3). From the stems of different alfalfa varieties, 21 bacterial strains were isolated (Supplementary Figure S5, Table S3). Molecular identification with primers 27F/1429R identified 21 species belonging to 4 genera. Among the total 50 bacterial strains obtained, 30 strains belonged to the domain Bacteria, phylum Bacillota, class Bacilli, order Bacillales, and family Bacillaceae. Another six strains belonged to the phylum Actinobacteria, class Actinomycetes. *V. alfalfae* was not isolated or identified from either the rhizosphere soil or the alfalfa stems. This confirms the accuracy of the sampling and verifies that all sampled plants were alfalfa plants not infected by *V. alfalfae*.

### Isolation rate of cultivable microorganisms

At a dilution concentration of 1 × 10^−2^ for the rhizosphere soil extracts of different varieties, 400–500 colonies were observed; at 1 × 10^−3^, 100–150 colonies; at 1 × 10^−4^, 20–40 colonies; and at 1 × 10^−5^, 3–12 colonies. Based on these results, the 1 × 10^−3^ dilution was determined to be the most suitable for bacterial colony counting (Supplementary Figure S6a).

The isolation rates of culturable bacteria from the rhizosphere soil varied among different alfalfa varieties. In the GN alfalfa variety, the isolation rates of *S. galilaeus* LYZ1124, *B. atrophaeus* LYZ1127, *B. atrophaeus* LYZ1129, *Peribacillus frigoritolerans* LYZ1144, *Streptomyces anulatus* LYZ1134, *Peribacillus simplex* LYZ1143, *Streptomyces europaeiscabiei* LYZ1138, *Schumannella luteola* LYZ1157, and *Brevibacillus laterosporus* LYZ1135 were also significantly higher than in other varieties. In the WL variety, the isolation rates of *Streptomyces bobili* LYZ1130, *Streptomyces microflavus* LYZ1132, *Peribacillus castrilensis* LYZ1145, and *Pseudoxanthomonas mexicana* LYZ1147 were significantly higher than in other varieties (Table 5).

**Table 5:** Culturable bacteria isolated from rhizosphere soil of different alfalfa varieties.

Strains	Gannong No.4	WL343HQ	Dryland	Magnum II	Xinmu No.1	Saranac
*Streptomyces galilaeus* LYZ1124	2.12 ± 0.93a	1.40 ± 0.56a	1.97 ± 0.67a	0.98 ± 0.41a	0.86 ± 0.47a	0.78 ± 0.55a
*Bacillus atrophaeus* LYZ1127	1.61 ± 0.77a	1.12 ± 0.62a	0.30 ± 0.30a	0.00 ± 0.00a	0.07 ± 0.07a	0.72 ± 0.50a
*Streptomyces bobili* LYZ1130	0.65 ± 0.41a	1.37 ± 0.69a	0.70 ± 0.46a	0.30 ± 0.30a	1.30 ± 0.49a	0.00 ± 0.00a
*Bacillus velezensis* LYZ1129	0.51 ± 0.38a	0.11 ± 0.11a	0.00 ± 0.00a	0.00 ± 0.00a	0.00 ± 0.00a	0.00 ± 0.00a
*Streptomyces microflavus* LYZ1132	5.02 ± 1.13ab	5.69 ± 0.94a	3.22 ± 0.83abc	1.48 ± 0.48c	3.00 ± 0.79bc	3.07 ± 0.78bc
*Peribacillus frigoritolerans* LYZ1144	0.61 ± 0.38a	0.19 ± 0.19a	0.00 ± 0.00a	0.23 ± 0.23a	0.39 ± 0.35a	0.16 ± 0.16a
*Streptomyces anulatus* LYZ1134	7.11 ± 0.92a	4.46 ± 0.90b	1.63 ± 0.71 cd	2.74 ± 0.87bc	1.80 ± 0.55 cd	0.00 ± 0.00d
*Peribacillus simplex* LYZ1143	6.99 ± 1.69a	5.58 ± 1.01ab	2.88 ± 0.95bc	1.33 ± 0.55c	2.66 ± 0.60c	0.85 ± 0.46c
*Peribacillus castrilensis* LYZ1145	3.92 ± 1.27ab	5.43 ± 0.98a	2.25 ± 0.90bc	2.91 ± 0.89abc	3.29 ± 0.66abc	1.02 ± 0.52c
*Pseudomonas migulae* LYZ1150	3.53 ± 0.98c	7.06 ± 1.02bc	11.82 ± 2.77ab	6.23 ± 0.62bc	14.99 ± 3.60a	6.80 ± 2.06bc
*Microbacterium foliorum* LYZ1137	7.09 ± 1.44bc	10.03 ± 1.33ab	14.17 ± 2.22a	13.87 ± 1.97a	8.18 ± 1.12bc	5.10 ± 1.31c
*Arthrobacter bambusae* LYZ1154	31.34 ± 3.00a	20.79 ± 1.36b	24.54 ± 3.77ab	31.99 ± 2.44c	33.53 ± 2.92c	31.02 ± 4.31c
*Microbacterium phyllosphaerae* LYZ1155	2.41 ± 0.71abc	4.59 ± 1.09ab	5.29 ± 0.75a	1.33 ± 0.47c	2.05 ± 0.52bc	4.40 ± 1.80ab
*Arthrobacter ruber* LYZ1156	2.62 ± 0.69bc	4.45 ± 1.16b	9.09 ± 2.20a	0.63 ± 0.37c	5.35 ± 1.35b	0.37 ± 0.30c
*Streptomyces europaeiscabiei* LYZ1138	5.61 ± 1.06a	4.55 ± 0.92ab	1.64 ± 0.54b	4.58 ± 0.67ab	1.87 ± 0.65b	4.03 ± 1.75ab
*Pseudoxanthomonas mexicana* LYZ1147	4.11 ± 1.01a	5.99 ± 2.75a	4.33 ± 1.02a	3.84 ± 0.55a	2.24 ± 0.81a	1.89 ± 1.05a
*Rhodococcus sovatensis* LYZ1151	4.22 ± 0.69ab	6.04 ± 0.61a	5.85 ± 1.24a	2.84 ± 0.80b	6.78 ± 1.45a	2.22 ± 0.87b
*Schumannella luteola* LYZ1157	2.57 ± 0.74a	0.98 ± 0.54ab	1.36 ± 0.46ab	2.20 ± 0.68a	1.70 ± 0.61ab	0.31 ± 0.31b
*Bacillus idriensis* LYZ1160	3.13 ± 0.96b	4.38 ± 0.75ab	2.37 ± 0.88b	6.43 ± 1.47a	1.46 ± 0.53b	3.88 ± 1.33ab
*Leucobacter chromiiresistens* LYZ1152	1.42 ± 0.63ab	1.58 ± 0.66ab	1.40 ± 0.97ab	3.65 ± 1.05a	3.86 ± 1.09a	0.00 ± 0.00b
*Bacillus altitudinis* LYZ1161	0.73 ± 0.50a	0.95 ± 0.44a	0.93 ± 0.61a	0.35 ± 0.35a	0.00 ± 0.00a	0.00 ± 0.00a
*Brevibacillus laterosporus* LYZ1135	1.39 ± 0.75a	0.34 ± 0.34ab	0.00 ± 0.00b	0.53 ± 0.30ab	0.07 ± 0.07b	0.84 ± 0.47ab
*Streptomyces umbrinus* LYZ1142	0.26 ± 0.26a	0.00 ± 0.00a	0.63 ± 0.43a	0.84 ± 0.63a	0.84 ± 0.28a	1.18 ± 0.70a
*Bacillus pumilus* LYZ1141	0.00 ± 0.00b	1.62 ± 0.84a	0.80 ± 0.44ab	1.05 ± 0.60ab	0.07 ± 0.07b	0.26 ± 0.26ab
*Pedobacter steynii* LYZ1162	0.00 ± 0.00a	0.00 ± 0.00a	0.00 ± 0.00a	0.77 ± 0.53a	0.47 ± 0.32a	0.00 ± 0.00a
*Pseudomonas arenae* LYZ1140	0.00 ± 0.00b	0.00 ± 0.00b	0.00 ± 0.00b	5.46 ± 1.71ab	2.06 ± 0.83b	10.08 ± 4.52a
*Glutamicibacter arilaitensis* LYZ1149	0.00 ± 0.00b	0.00 ± 0.00b	0.00 ± 0.00b	0.00 ± 0.00b	0.00 ± 0.00b	9.20 ± 4.63a
*Pseudomonas baetica* LYZ1139	0.00 ± 0.00b	0.00 ± 0.00b	0.00 ± 0.00b	0.00 ± 0.00b	0.00 ± 0.00b	11.33 ± 6.07a
*Stenotrophomonas maltophilia* LYZ1158	0.00 ± 0.00a	0.00 ± 0.00a	0.00 ± 0.00a	0.14 ± 0.14a	0.00 ± 0.00a	0.00 ± 0.00a

Different letters within the same line indicate significant differences in isolation rate of rhizosphere soil among different varieties (*p* < 0.05).

The isolation rates of culturable bacteria in the stems of different alfalfa varieties varied. In the GN variety, the isolation rates of *B. velezensis* LYZ1126, *B. subtilis* LYZ1128, and *Peribacillus frigoritolerans* LYZ1146 were significantly higher than those in other varieties. Conversely, in the WL variety, the isolation rates of *B. amyloliquefaciens* LYZ1125, *Bacillus australimaris* LYZ1164, and *Bacillus thuringiensis* LYZ1168 were significantly higher as well (Table 6).

**Table 6:** Culturable bacteria isolated from plant stalks of different alfalfa varieties.

Strains	Gannong No.4	WL343HQ	Dryland	Magnum II	Xinmu No.1	Saranac
*Bacillus australimaris* LYZ1164	0.00 ± 0.00a	13.67 ± 11.70a	0.00 ± 0.00a	0.00 ± 0.00a	0.00 ± 0.00a	10.00 ± 10.00a
*Bacillus subtilis* LYZ1128	16.69 ± 16.69a	1.67 ± 1.67a	6.82 ± 6.82a	14.29 ± 14.29a	0.00 ± 0.00a	10.71 ± 10.71a
*Priestia aryabhattai* LYZ1131	3.33 ± 3.33a	3.33 ± 3.33a	7.64 ± 7.64a	10.00 ± 10.00a	0.00 ± 0.00a	0.00 ± 0.00a
*Bacillus pumilus* LYZ1148	10.14 ± 6.16b	17.33 ± 5.31ab	33.15 ± 5.07a	2.86 ± 2.86b	11.67 ± 7.26b	9.00 ± 5.57b
*Solibaccillus silvestris* LYZ1159	0.00 ± 0.00b	15.00 ± 7.64a	1.67 ± 1.67b	0.00 ± 0.00b	0.00 ± 0.00b	2.50 ± 2.50b
*Bacillus thuringiensis* LYZ1168	2.67 ± 2.67a	25.00 ± 19.36a	1.82 ± 1.82a	11.43 ± 9.74a	6.67 ± 6.67a	9.71 ± 6.10a
*Bacillus amyloliquefaciens* LYZ1125	0.00 ± 0.00a	6.67 ± 6.67a	0.00 ± 0.00a	0.00 ± 0.00a	0.00 ± 0.00a	0.00 ± 0.00a
*Bacillus safensis* LYZ1136	1.25 ± 1.25ab	5.00 ± 3.33ab	9.30 ± 3.18ab	12.38 ± 7.62a	0.00 ± 0.00b	0.00 ± 0.00b
*Bacillus gibsonii* LYZ1163	0.00 ± 0.00a	1.67 ± 1.67a	0.00 ± 0.00a	0.00 ± 0.00a	0.00 ± 0.00a	0.00 ± 0.00a
*Bacillus velezensis* LYZ1126	14.11 ± 14.11ab	0.00 ± 0.00b	12.79 ± 12.79ab	23.10 ± 23.10a	0.00 ± 0.00b	11.50 ± 11.50ab
*Bacillus aerius* LYZ1171	0.00 ± 0.00a	0.00 ± 0.00a	1.82 ± 1.82a	5.00 ± 5.00a	0.00 ± 0.00a	0.00 ± 0.00a
*Peribacillus frigoritolerans* LYZ1146	6.11 ± 4.84a	0.00 ± 0.00a	0.00 ± 0.00a	0.00 ± 0.00a	0.00 ± 0.00a	0.00 ± 0.00a
*Bacillus simplex* LYZ1174	17.61 ± 17.61a	3.33 ± 3.33a	7.27 ± 7.27a	8.10 ± 8.10a	30.00 ± 30.00a	2.86 ± 2.86a
*Bacillus toyonensis* LYZ1170	6.25 ± 4.84a	0.00 ± 0.00a	0.00 ± 0.00a	1.43 ± 1.43a	0.00 ± 0.00a	0.00 ± 0.00a
*Bacillus mobilis* LYZ1173	3.92 ± 3.92a	0.00 ± 0.00a	8.79 ± 8.79a	0.00 ± 0.00a	8.33 ± 8.33a	2.50 ± 2.50a
*Peribacillus castrilensis* LYZ1153	0.00 ± 0.00a	4.00 ± 4.00a	0.00 ± 0.00a	1.43 ± 1.43a	5.00 ± 5.00a	0.00 ± 0.00a
*Bacillus licheniformis* LYZ1166	0.00 ± 0.00b	0.00 ± 0.00b	0.00 ± 0.00b	0.00 ± 0.00b	0.00 ± 0.00b	13.36 ± 13.36a
*Bacillus inaquosorum* LYZ1133	0.00 ± 0.00a	0.00 ± 0.00a	0.00 ± 0.00a	0.00 ± 0.00a	0.00 ± 0.00a	2.50 ± 2.50a
*Bacillus idriensis* LYZ1172	0.00 ± 0.00a	0.00 ± 0.00a	0.00 ± 0.00a	0.00 ± 0.00a	0.00 ± 0.00a	22.50 ± 19.35a
*Paenibacillus harenae* LYZ1167	0.00 ± 0.00a	0.00 ± 0.00a	0.00 ± 0.00a	0.00 ± 0.00a	0.00 ± 0.00a	2.86 ± 2.86a
*Bacillus altitudinis* LYZ1165	15.56 ± 15.56ab	3.33 ± 3.33b	5.30 ± 5.30b	10.00 ± 10.00ab	35.00 ± 35.00a	0.00 ± 0.00b

Different letters within the same line indicate significant differences in isolation rate of plant stalks among different varieties (*p* < 0.05).

The isolation rates of culturable fungi in the rhizosphere soil varied among different alfalfa varieties. *Boeremia foveata* ZLL1 showed higher isolation rates in the WL and JN varieties, while *Phoma medicaginis* ZLL2 exhibited a significantly higher isolation rate in the SA variety compared to others. The isolation rate of *Clonostachys rosea* ZLL5 was significantly higher in GN and SA than in other varieties, and *Mortierella alpina* ZLL6 had a significantly higher isolation rate in XM compared to other varieties (Supplementary Figure S6b).

### Inhibition rate of culturable bacteria against *V. Alfalfae*

**Figure 6:**
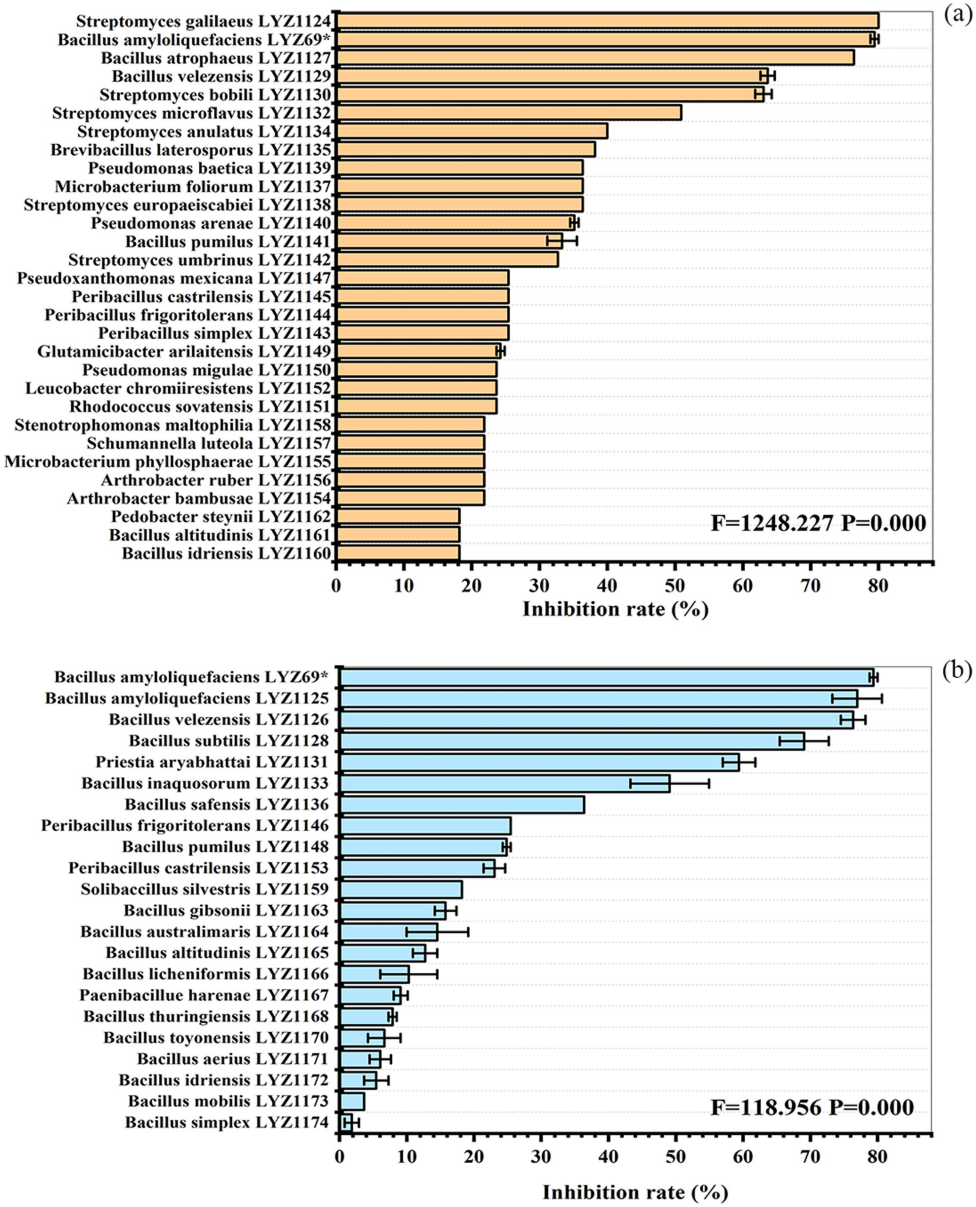
Inhibition rates of 51 bacterial strains from rhizosphere soil **(a)** and stem **(b)** against *V. alfalfae* LYZ0257. “*” denotes the control strain.

There were significant differences (*p* < 0.05) in the inhibition rates of different bacterial strains against *V. alfalfae* LYZ0257 (Figure 6, Supplementary Figure S7). The control strain, *B. amyloliquefaciens* LYZ69, exhibited an inhibition rate of 79%. The strains with inhibition rates greater than 60% included *S. galilaeus* LYZ1124, *B. amyloliquefaciens* LYZ1125, *B. velezensis* LYZ1126, *B. atrophaeus* LYZ1127, *B. subtilis* LYZ1128, *B. atrophaeus* LYZ1129, and *S. bobili* LYZ1130. Twelve strains showed inhibition rates between 30 and 60%, while 31 strains had inhibition rates below 30% (Figure 6). Among them, the inhibition rates of *S. galilaeus* LYZ1124, *B. amyloliquefaciens* LYZ1125, *B. velezensis* LYZ1126, *B. atrophaeus* LYZ1127, and *B. subtilis* LYZ1128 did not significantly differ from the control strain (Figure 6).

### Physical and chemical properties suitable for bacterial growth

Of the 50 bacterial strains tested for physicochemical properties, 47 exhibited proteolytic activity, 38 demonstrated nitrogen-fixing capacity, 38 showed potassium solubilizing ability, 9 were capable of solubilizing organic phosphorus, and 5 could solubilize inorganic phosphorus (Table 7). *B. velezensis* LYZ1126 and *B. atrophaeus* LYZ1127 displayed significantly stronger proteolytic activity than other strains, making them priority candidates for further research (Table 7). Based on both antibacterial efficacy and physicochemical characteristics, *B. amyloliquefaciens* LYZ1125, *B. velezensis* LYZ1126, *B. atrophaeus* LYZ1127, and *B. subtilis* LYZ1128 were selected as potential superior microbial resources for controlling alfalfa Verticillium wilt. These strains will be used in subsequent experiments to verify the biocontrol effects of *Bacillus* strains against this disease.

**Table 7:** Physicochemical properties of 51 bacterial strains.

Strain	Nitrogen fixation capacity	Potassium dissolving capacity	Capacity for dissolving organophosphorus compounds (D/d)	Ability to dissolve inorganic phosphorus (D/d)	Protein dissolution capacity (D/d)
*Streptomyces galilaeus* LYZ1124	−	−	4.34 ± 2.58	0.00 ± 0.00	3.49 ± 0.60
*Bacillus amyloliquefaciens* LYZ0069*	+	+	0.00 ± 0.00	0.00 ± 0.00	3.03 ± 0.22
*Bacillus amyloliquefaciens* LYZ1125	+	+	0.00 ± 0.00	0.00 ± 0.00	6.10 ± 0.45
*Bacillus velezensis* LYZ1126	+	+	0.00 ± 0.00	0.00 ± 0.00	13.30 ± 1.64
*Bacillus atrophaeus* LYZ1127	+	+	0.00 ± 0.00	0.00 ± 0.00	13.11 ± 2.00
*Bacillus subtilis* LYZ1128	+	+	0.00 ± 0.00	0.00 ± 0.00	9.08 ± 2.85
*Bacillus atrophaeus* LYZ1129	+	+	0.00 ± 0.00	0.00 ± 0.00	9.19 ± 0.72
*Streptomyces bobili* LYZ1130	−	−	0.00 ± 0.00	0.00 ± 0.00	4.25 ± 0.93
*Priestia aryabhattai* LYZ1131	+	+	0.00 ± 0.00	1.06 ± 0.34	2.80 ± 0.18
*Streptomyces microflavus* LYZ1132	+	−	7.86 ± 4.65	0.00 ± 0.00	4.68 ± 1.19
*Bacillus inaquosorum* LYZ1133	+	+	0.00 ± 0.00	12.68 ± 0.42	6.84 ± 1.17
*Streptomyces anulatus* LYZ1134	+	+	0.00 ± 0.00	22.06 ± 4.05	9.16 ± 1.31
*Brevibacillus laterosporus* LYZ1135	+	+	0.00 ± 0.00	0.00 ± 0.00	9.65 ± 0.82
*Bacillus safensis* LYZ1136	+	+	0.00 ± 0.00	0.00 ± 0.00	9.86 ± 2.10
*Microbacterium foliorum* LYZ1137	+	+	0.00 ± 0.00	0.00 ± 0.00	2.80 ± 0.16
*Streptomyces europaeiscabiei* LYZ1138	−	−	0.00 ± 0.00	0.00 ± 0.00	3.94 ± 0.90
*Pseudomonas baetica* LYZ1139	+	+	0.00 ± 0.00	0.00 ± 0.00	8.12 ± 3.01
*Pseudomonas arenae* LYZ1140	+	+	4.45 ± 2.81	0.00 ± 0.00	5.53 ± 1.22
*Bacillus pumilus* LYZ1141	+	+	12.15 ± 6.22	0.00 ± 0.00	5.75 ± 1.57
*Streptomyces umbrinus* LYZ1142	+	+	2.75 ± 0.81	0.00 ± 0.00	4.35 ± 0.73
*Peribacillus simplex* LYZ1143	+	+	0.00 ± 0.00	0.00 ± 0.00	4.41 ± 0.41
*Peribacillus simplex* LYZ1144	−	+	0.00 ± 0.00	0.00 ± 0.00	2.89 ± 0.24
*Peribacillus castrilensis* LYZ1145	+	+	0.00 ± 0.00	16.83 ± 3.37	2.36 ± 0.14
*Peribacillus frigoritolerans* LYZ1146	−	−	0.00 ± 0.00	0.00 ± 0.00	6.35 ± 1.17
*Pseudoxanthomonas mexicana* LYZ1147	−	−	0.00 ± 0.00	0.00 ± 0.00	3.37 ± 0.17
*Bacillus pumilus* LYZ1148	+	+	3.42 ± 0.42	0.00 ± 0.00	3.47 ± 0.19
*Glutamicibacter arilaitensis* LYZ1149	+	+	0.00 ± 0.00	0.00 ± 0.00	2.66 ± 0.26
*Pseudomonas migulae* LYZ1150	+	+	3.50 ± 0.43	0.00 ± 0.00	0.00 ± 0.00
*Rhodococcus sovatensis* LYZ1151	+	+	13.12 ± 4.56	4.93 ± 0.39	3.23 ± 0.16
*Leucobacter chromiiresistens* LYZ1152	−	−	0.00 ± 0.00	0.00 ± 0.00	9.92 ± 3.41
*Peribacillus castrilensis* LYZ1153	+	+	0.00 ± 0.00	0.00 ± 0.00	6.43 ± 1.36
*Arthrobacter bambusae* LYZ1154	−	−	0.00 ± 0.00	0.00 ± 0.00	3.88 ± 0.24
*Microbacterium phyllosphaerae* LYZ1155	−	−	0.00 ± 0.00	0.00 ± 0.00	5.13 ± 0.98
*Arthrobacter ruber* LYZ1156	−	−	0.00 ± 0.00	0.00 ± 0.00	6.10 ± 1.19
*Schumannella luteola* LYZ1157	−	−	0.00 ± 0.00	0.00 ± 0.00	1.69 ± 1.07
*Stenotrophomonas maltophilia* LYZ1158	+	+	0.00 ± 0.00	0.00 ± 0.00	3.45 ± 0.16
*Solibaccillus silvestris* LYZ1159	+−	+	0.00 ± 0.00	0.00 ± 0.00	0.00 ± 0.00
*Bacillus idriensis* LYZ1160	+	+	15.51 ± 5.42	0.00 ± 0.00	3.50 ± 0.27
*Bacillus altitudinis* LYZ1161	+	+	0.00 ± 0.00	0.00 ± 0.00	3.43 ± 0.17
*Pedobacter steynii* LYZ1162	−	−	0.00 ± 0.00	0.00 ± 0.00	2.59 ± 0.19
*Bacillus gibsonii* LYZ1163	+	+	0.00 ± 0.00	0.00 ± 0.00	0.44 ± 0.44
*Bacillus australimaris* LYZ1164	+	+	0.00 ± 0.00	0.00 ± 0.00	2.78 ± 0.23
*Bacillus altitudinis* LYZ1165	+	+	0.00 ± 0.00	0.00 ± 0.00	2.53 ± 1.15
*Bacillus licheniformis* LYZ1166	+	+	0.00 ± 0.00	0.00 ± 0.00	0.82 ± 0.82
*Paenibacillus harenae* LYZ1167	+	+	0.00 ± 0.00	0.00 ± 0.00	9.24 ± 3.04
*Bacillus thuringiensis* LYZ1168	+	+	0.00 ± 0.00	0.00 ± 0.00	6.96 ± 1.63
*Bacillus toyonensis* LYZ1170	+	+	0.00 ± 0.00	0.00 ± 0.00	0.00 ± 0.00
*Bacillus aerius* LYZ1171	+	+	0.00 ± 0.00	0.00 ± 0.00	2.53 ± 0.65
*Bacillus idriensis* LYZ1172	+	+	0.00 ± 0.00	0.00 ± 0.00	7.66 ± 1.51
*Bacillus mobilis* LYZ1173	+	+	0.00 ± 0.00	0.00 ± 0.00	3.86 ± 0.76
*Bacillus simplex* LYZ1174	+	+	0.00 ± 0.00	0.00 ± 0.00	3.21 ± 0.33

“+” indicates that this feature is available, while “−” indicates that it is not available. “*” denotes the control strain.

## Discussion

This study found that the alpha diversity of rhizosphere bacterial communities differed only in specific indices among alfalfa varieties with varying resistance levels. Notably, the Simpson diversity index was significantly higher in the resistant variety GN than in the susceptible variety SA, while richness indices (Chao1, ACE) and the Shannon index showed no significant variation. This pattern contrasts with some reports on disease-resistant wheat varieties, where increases were observed across multiple diversity indices (Li H. et al., 2023). The discrepancy may stem from differences in crop species, pathogen types, soil microenvironments, and the specificity of plant–microbe interactions. Our results suggest that in the alfalfa Verticillium wilt system, resistant varieties affect rhizosphere bacterial diversity in a selective manner rather than uniformly enhancing all aspects of diversity. This view aligns with Acuna et al. (2023)and Li et al. (2020), who emphasized the complex, multifactorial influence of plant genotype on the microbiome. In contrast, fungal alpha diversity did not differ significantly among varieties, consistent with observations in wheat (Li H. et al., 2023), indicating that rhizosphere fungal communities may be less responsive to variations in plant resistance than bacterial communities.

In this study, the rhizosphere soil of healthy plants from the highly resistant alfalfa variety Gannong No.4 exhibited higher relative abundances of the genera *Devosia* and *Novosphingobium* compared to the more susceptible varieties Xinmu No.1 and Saranac, implying a potential association between the rhizosphere enrichment of these two genera and alfalfa Verticillium wilt resistance. Previous research has shown that *Novosphingobium* can induce systemic resistance in pepper against bacterial spot disease (Mi-Seon et al., 2012) and inhibit the growth of soil-borne pathogens such as *Sclerotium rolfsii* and *Leptosphaerulina arachidicola* (Zhang et al., 2021). Similarly, *Devosia* has been reported to induce the degradation of deoxynivalenol mycotoxins and serve as biocontrol agents against *Fusarium* crown rot in wheat (He et al., 2024), with its functional characteristics closely related to the inhibition of pathogenic fungal activity and the improvement of plant disease resistance. These reported functional traits of *Devosia* and *Novosphingobium* provide important indirect evidence for their potential role in mediating alfalfa resistance to Verticillium wilt, indicating that alfalfa varieties with strong resistance to Verticillium wilt may actively recruit these beneficial microorganisms in the rhizosphere to enhance their resistance against the pathogenic fungus *V. alfalfae*. Furthermore, studies have indicated that successful pathogen invasion in plants may be facilitated by host nutrient deficiencies, and nutrient competition significantly drives plant–microbe interactions (Cheng et al., 2019). Nitrogen, phosphorus, and potassium are essential macronutrients that enhance crop resistance to biotic stress (Qu et al., 2025). However, inappropriate levels of these soil nutrients can compromise plant defenses while favoring the establishment of soil-borne pathogens (Qu et al., 2025). Soil organic matter serves as a nutrient source for soil microorganisms (Sheng-zhe et al., 2018), and its content has been shown to be strongly associated with soil-borne diseases (Qu et al., 2025). Therefore, investigating soil physicochemical properties is crucial for controlling soil-borne diseases. In our study, the promising biocontrol genera *Devosia* and *Novosphingobium* were positively correlated with soil total nitrogen (TN), available potassium (AK), organic matter (SOM), and pH. Thus, future strategies for managing alfalfa Verticillium wilt could include managing soil TN, AK, and SOM levels to promote the colonization of these beneficial bacteria, thereby enhancing plant defense against *V. alfalfae*. Of course, future studies should adopt spatially explicit sampling designs to more rigorously disentangle the effects of environment, space, and host genotype.

Soil microbial diversity is a critical driver of agricultural ecosystem function and health (Zhang et al., 2025). A study demonstrated that the rhizosphere soil of highly resistant wheat varieties harbors greater microbial diversity than that of susceptible varieties, with higher ACE, Chao1, Simpson, and Shannon indices observed in the resistant cultivars (Li H. et al., 2023). Another study also reported that wheat varieties resistant to wheat mosaic virus disease exhibited higher rhizosphere bacterial alpha diversity indices compared to susceptible varieties (Wu et al., 2021). However, our study found that only the Simpson index of the highly resistant alfalfa cultivar Gannong No.4 was significantly greater than that of the susceptible cultivar Saranac, which is partially inconsistent with the above-mentioned results. This inconsistency may be caused by variations in host species-specific interactions, distinct pathogenic microbes, and different edaphic conditions across experimental systems.

Recent advances in biotechnology have enabled the development of novel strategies for controlling soil-borne diseases. A prominent approach involves constructing synthetic microbial consortia (SynComs) from beneficial microbial communities, including plant growth-promoting rhizobacteria (Nagrale et al., 2023), core microbiomes (Neu et al., 2021), and keystone taxa (Gibbons, 2020), for sustainable pathogen management (Agrahari et al., 2020; Fierer, 2017). In our study, LefSe analysis revealed that the Bacillota, Bacilli, Bacillales, Bacillaceae, and Actinobacteria were key bacterial taxa in the rhizosphere of alfalfa varieties with differing resistance levels. These bacterial groups should be prioritized in subsequent screenings for biocontrol agents, as they represent a promising source of potential biocontrol resources.

*Bacillus* species, which are widely distributed in nature, are important beneficial microorganisms capable of producing highly stress-resistant spores, a diverse array of antibiotics, and enzymes (Wang, 2013). To date, numerous *Bacillus* strains have been developed as agents for plant growth promotion, insect control, and bactericidal applications (Xu et al., 2024). Among rhizobacteria, *Bacillus* is regarded as one of the most effective biocontrol agents (Chen, 2023), with demonstrated efficacy against diseases in a variety of crops, including alfalfa (*M. sativa*), wheat (*Triticum aestivum*), and maize (*Zea mays*) (Wang, 2013). As of now, over 300 species within the genus *Bacillus* have been reported (Parte et al., 2020), with common species used for disease control including *B. amyloliquefaciens*, *B. subtilis*, and *B. velezensis* (Singh and Shyu, 2024).

*Streptomyces* are a group of gram-positive bacteria whose cell walls primarily consist of peptidoglycan. They are widely distributed in terrestrial, marine, and internal plant environments, though soil remains the primary source for *Streptomyces* isolation(Lv et al., 2025). Research indicates that *Streptomyces* from acidic soils produce antimicrobials primarily active against filamentous fungi (Zakalyukina and Zenova, 2007). *Streptomyces* not only inhabit various plant tissues but also produce diverse antifungal metabolites, underscoring their broad potential in suppressing fungal diseases (Lv et al., 2025).

Our analysis identified the phylum Bacillota (encompassing class Bacillales and family Bacillaceae) and the phylum Actinobacteria as key bacterial groups in the rhizosphere of disease-resistant alfalfa varieties. Consistent with this finding, we isolated 50 strains from various alfalfa cultivars, of which 60% belonged to Bacillus and 12% to Streptomyces, supporting the results of these genera. Additionally, plate antagonism assays of the 50 strains identified five isolates, including *S. galilaeus* LYZ1124, *B. amyloliquefaciens* LYZ1125, *B. velezensis* LYZ1126, *B. atrophaeus* LYZ1127, and *B. subtilis* LYZ1128 with inhibition rates exceeding 60%. The difference in their inhibition rates compared to the control strain *B. amyloliquefaciens* LYZ0069 was not significant. They therefore constitute a primary source for screening biocontrol agents against alfalfa Verticillium wilt.

Biocontrol agents with promising application prospects not only secrete various secondary metabolites to directly inhibit pathogens but also possess functions such as phosphorus solubilization, potassium dissolution, nitrogen fixation, and proteolysis (Shan, 2025). Phosphorus and potassium are essential elements for promoting plant growth (Ruan et al., 2025). However, over 80% of the phosphorus in soil is fixed through binding with metal ions such as Fe^3+^, Fe^2+^, and Al^3+^, making it difficult for plants to absorb and utilize directly (Zhou et al., 2021). Phosphorus-solubilizing microorganisms can effectively decompose fixed phosphorus in soil using bioenzymes and organic acids (Chen et al., 2006), thereby increasing the availability of phosphorus for plant uptake. Ruchi et al. also demonstrated that phosphorus plays no significant role in plant disease resistance; however, elevated phosphorus levels may enhance plant susceptibility to pathogen invasion (Ruchi et al., 2022). Additionally, approximately 70% of China’s arable land is potassium-deficient (Wu et al., 2016). Potassium-dissolving bacteria in the rhizosphere not only promote plant growth but also stimulate crops to secrete substances that enhance stress resistance and disease resistance (Sturzav and Nowak, 2000). The ability of certain soil bacteria (*Bacillus*) to secrete proteases for degrading environmental proteins into absorbable nutrients ultimately leads to increased activities of catalase, sucrase, phosphatase, and urease in the rhizosphere (Xu, 2023). As an essential macronutrient, nitrogen is utilized by crops through the direct uptake of inorganic forms to sustain organic structures and promote growth (Wang et al., 2025). Among the 50 bacterial strains tested, 96% exhibited proteolytic activity, 90% demonstrated nitrogen-fixing capability, and 76% solubilized potassium, whereas only 1 to 2% solubilized phosphorus. This functional prevalence indicates a widespread potential for plant growth promotion. However, since most *Streptomyces* strains lacked these key agronomic traits, we can select *B. amyloliquefaciens* LYZ1125, *B. velezensis* LYZ1126, *B. atrophaeus* LYZ1127, and *B. subtilis* LYZ11284 for biological control verification against alfalfa Verticillium wilt.

## Conclusion

The rhizosphere soil of alfalfa varieties highly resistant to Verticillium wilt may recruit beneficial microbiota against the pathogen *V. alfalfae*.

## Data Availability

All clean sequencing data from this project of rhizosphere soil are available in the NCBI sequence read archive (SRA) database under bioproject PRJNA1223621, and the detailed information is provided in Supplementary Tables S1, S2. Culturable bacteria from rhizosphere soil 16S sequencing data are available under bioprojects PV110947 to PV110975, and culturable bacteria from alfalfa stems 16S sequencing data are available under bioprojects PV110989 to PV111008.
