# Supplementary material for: Effect of alfalfa varieties with different resistance to alfalfa Verticillium wilt on microbial communities in rhizosphere soil and plants
Source: Front Microbiol. 2026 May 19;17:1739219. doi: 10.3389/fmicb.2026.1739219 (PMC13226603; doi:10.3389/fmicb.2026.1739219)
Supplement: Supplementary file 1 [file Data_Sheet_1.PDF]

Supplement material

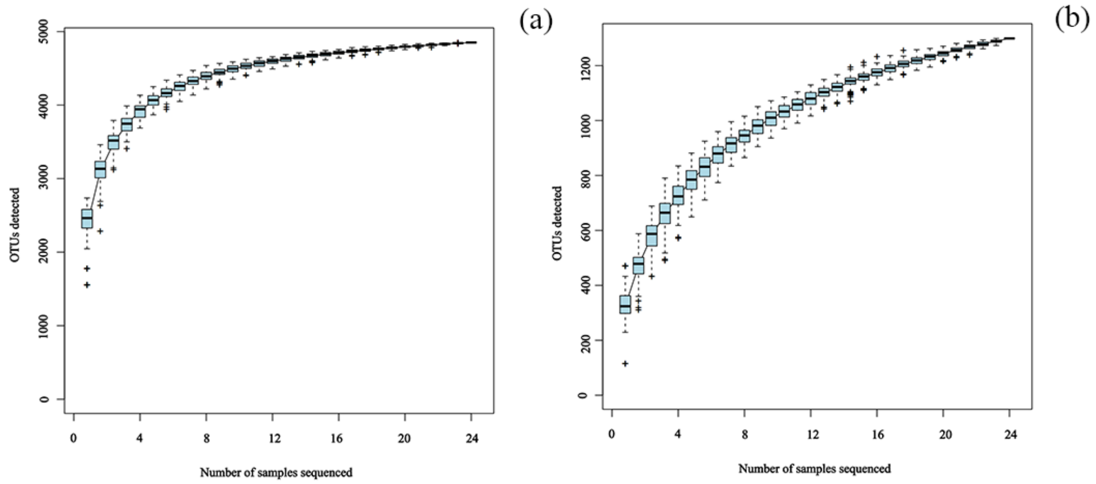

Figure S1. Cumulative curves of bacterial **(a)** and fungal **(b)** OTUs detected with increasing number of samples sequenced.

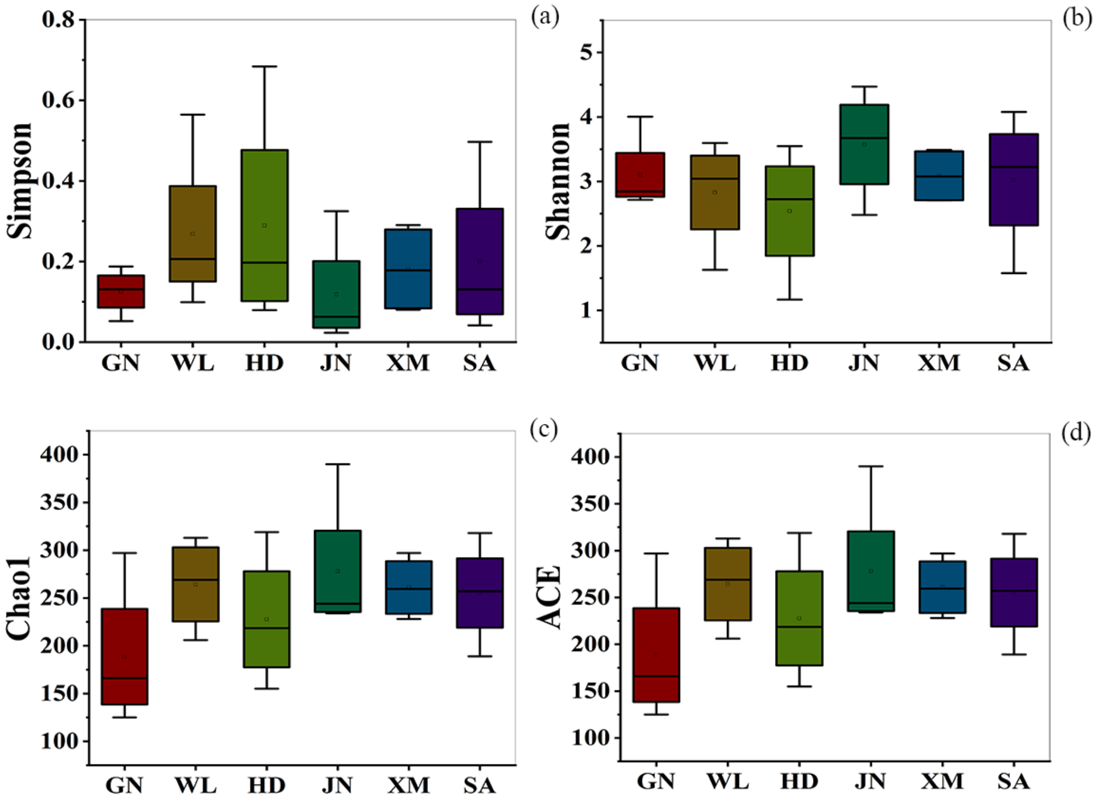

Figure S2. The alpha diversity index of the soil fungi communities under different varieties. (**a**) Simpson diversity index. (**b**) Shannon diversity index. (**c**) Chao1 richness index. (**d**) ACE richness index. GN: Gannong No.4 alfalfa variety; WL: WL343HQ alfalfa variety; HD: Dryland alfalfa variety; JN: Magnum II; XM: Xinmu No.1 alfalfa variety; SA: Saranac alfalfa variety.

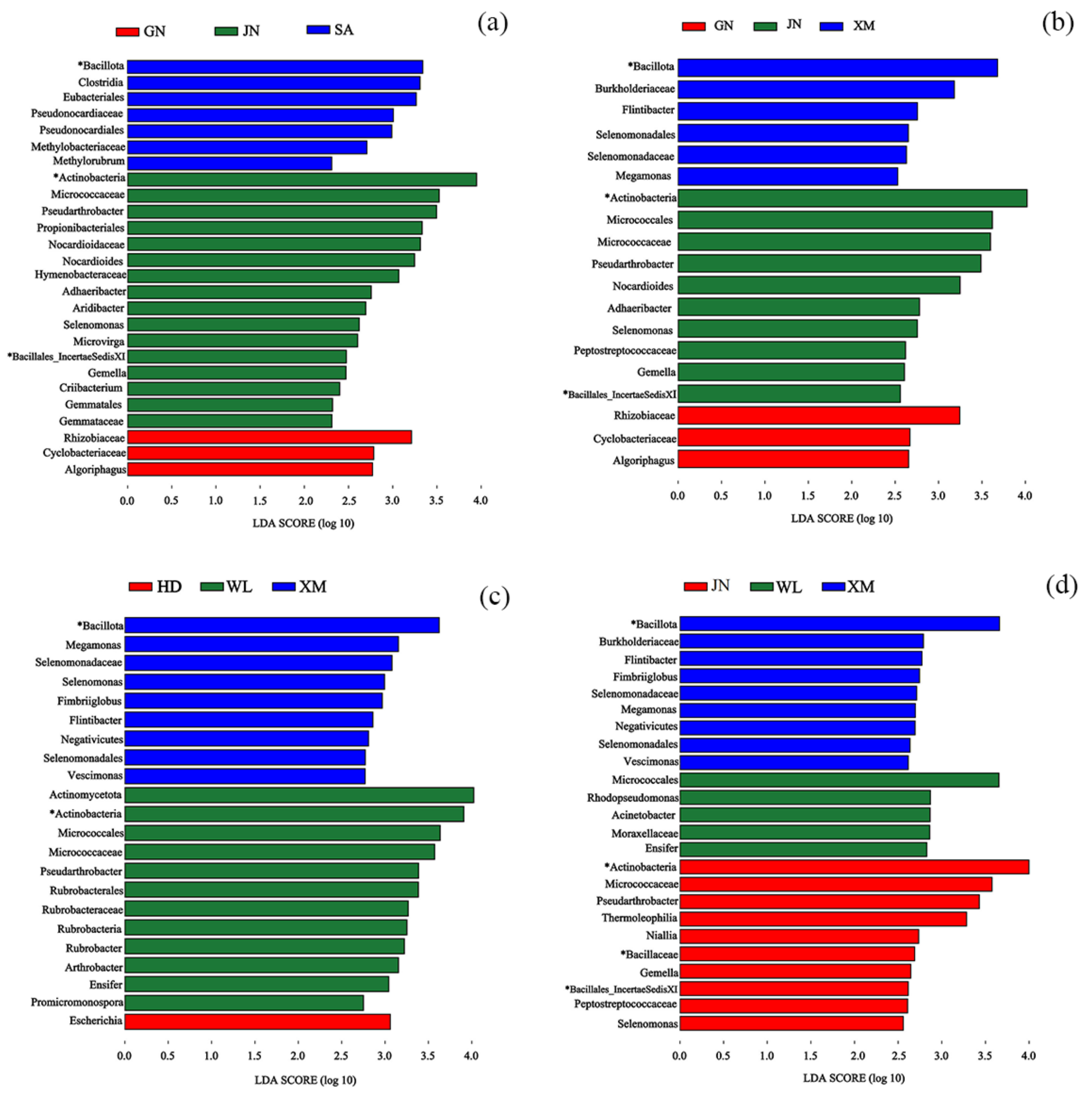

Figure S3. LDA Score Bar Plot shows the statistically significant and biologically relevant differential microbial lineages and their LDA scores under different alfalfa varieties. (a) Statistically significant and biologically relevant differential bacterial lineages and their LDA scores under GN, JN, and SA treatments; (b) Statistically significant and biologically relevant differential bacterial lineages and their LDA scores under GN, JN, and XM treatments; (c) Statistically significant and biologically relevant differential bacterial lineages and their LDA scores under HD, WL, and XM treatments; (d) Statistically significant and biologically relevant differential bacterial lineages and their LDA scores under WL, JN, and XM treatments. GN: Gannong No.4 alfalfa variety; WL: WL343HQ alfalfa variety; HD: Dryland alfalfa variety; JN: Magnum II; XM: Xinmu No.1 alfalfa variety; SA: Saranac alfalfa variety.

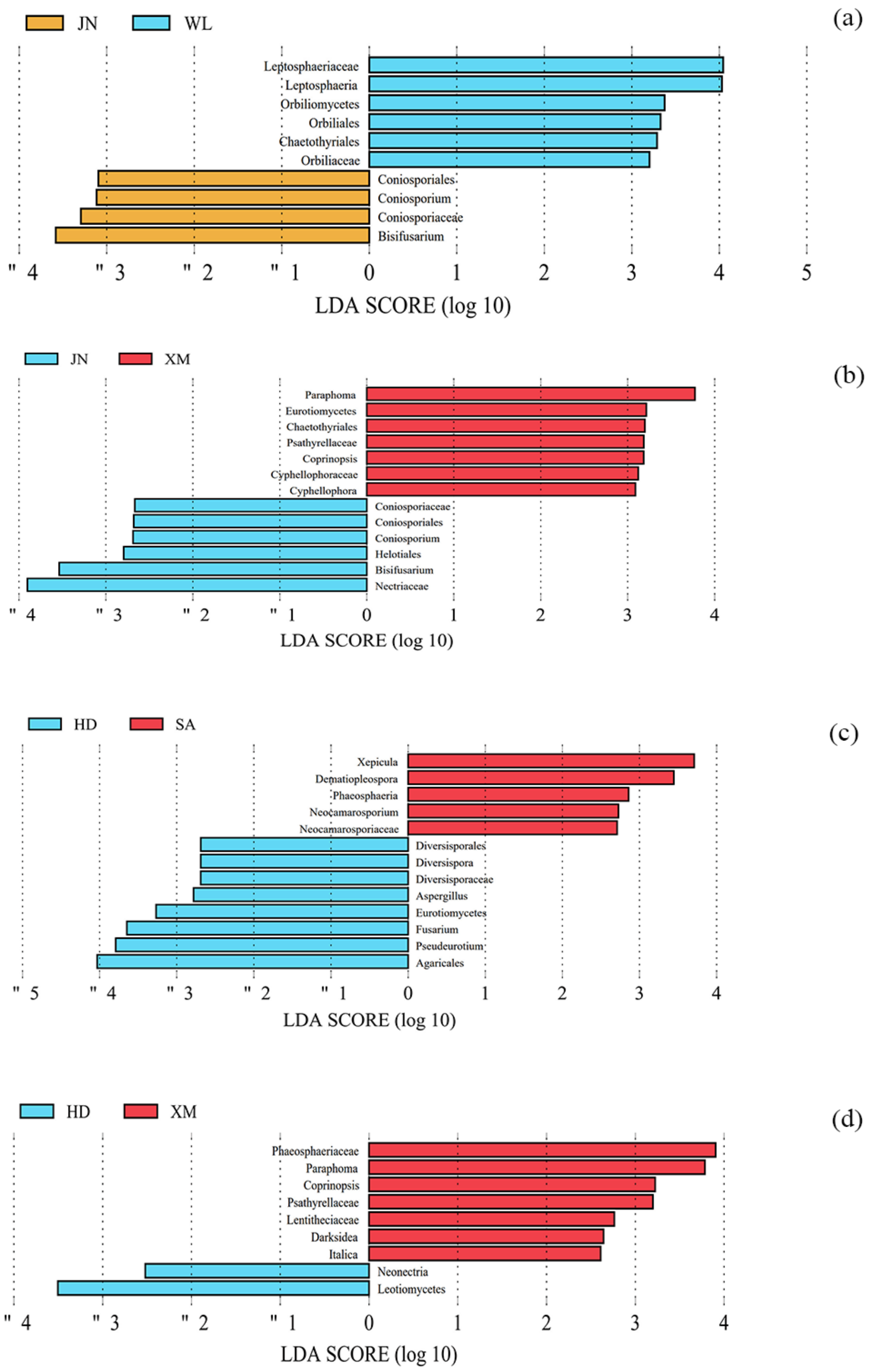
JN
WL
(a)
Leptosphaeriaceae
Leptosphaeria
Orbiliomycetes
Orbiliales
Chaetothyriales
Orbiliaceae
Coniosporiales
Coniosporium
Coniosporiaceae
Bisifusarium
" 4
" 3
" 2
" 1
0
1
2
3
4
5
LDA SCORE (log 10)
JN
XM
(b)
Paraphoma
Eurotiomycetes
Chaetothyriales
Psathyrellaceae
Coprinopsis
Cyphellophoraceae
Cyphellophora
Coniosporiaceae
Coniosporiales
Coniosporium
Helotiales
Bisifusarium
Nectriaceae
" 4
" 3
" 2
" 1
0
1
2
3
4
LDA SCORE (log 10)
HD
SA
(c)
Xepicula
Dematiopleospora
Phaeosphaeria
Neocamarosporium
Neocamarosporiaceae
Diversisporales
Diversispora
Diversisporaceae
Aspergillus
Eurotiomycetes
Fusarium
Pseudeurotium
Agaricales
" 5
" 4
" 3
" 2
" 1
0
1
2
3
4
LDA SCORE (log 10)
HD
XM
(d)
Phaeosphaeriaceae
Paraphoma
Coprinopsis
Psathyrellaceae
Lentitheciaceae
Darksidea
Italica
Neonectria
Leotiomycetes
" 4
" 3
" 2
" 1
0
1
2
3
4
LDA SCORE (log 10)

Figure S4. LDA Score Bar Plot shows the statistically significant and biologically relevant differential microbial lineages and their LDA scores under different alfalfa varieties. (a) Statistically significant and biologically relevant differential fungi lineages and their LDA scores under WL and JN, JN and XM, HD and SA, HD and XM treatments; GN: Gannong No.4 alfalfa variety; WL: WL343HQ alfalfa variety; HD: Dryland alfalfa variety; JN: Magnum II; XM: Xinmu No.1 alfalfa variety; SA: Saranac alfalfa variety.

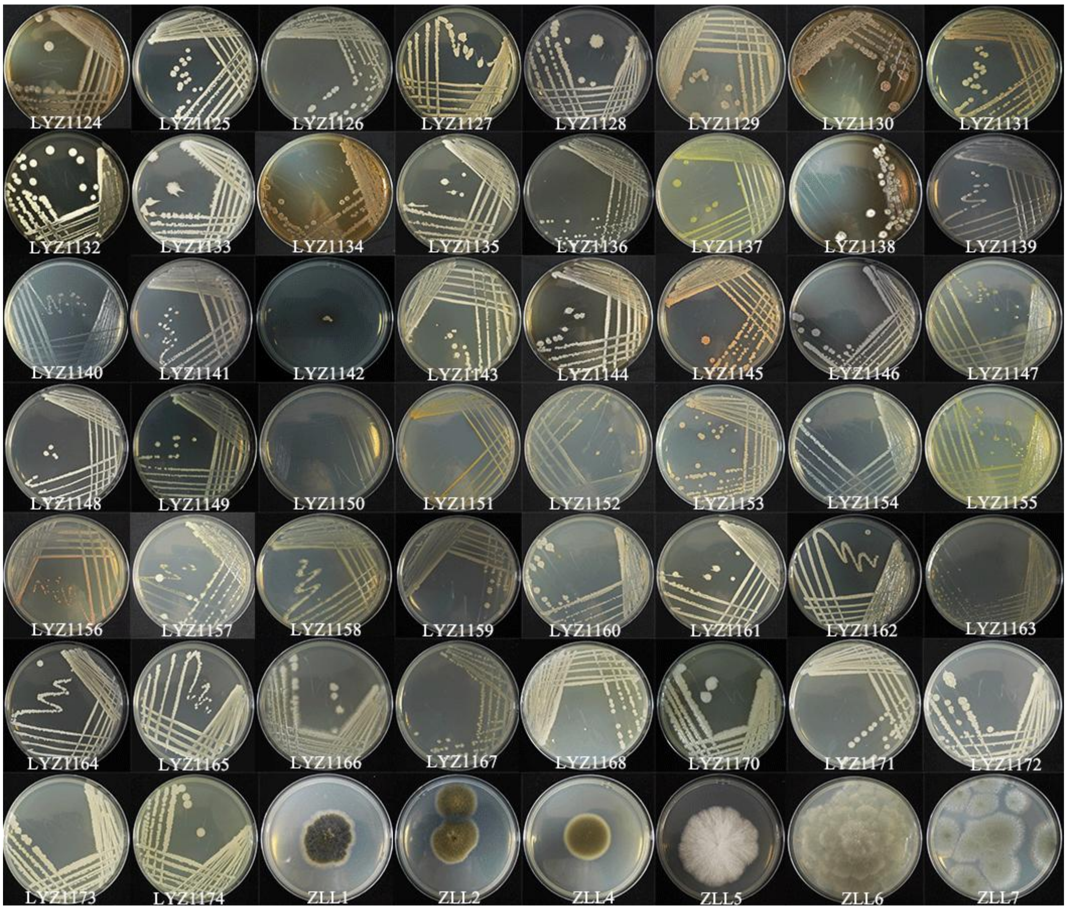

Figure S5. Microbial colonies can be isolated and cultured from rhizosphere soil and plant stalks of different alfalfa varieties.

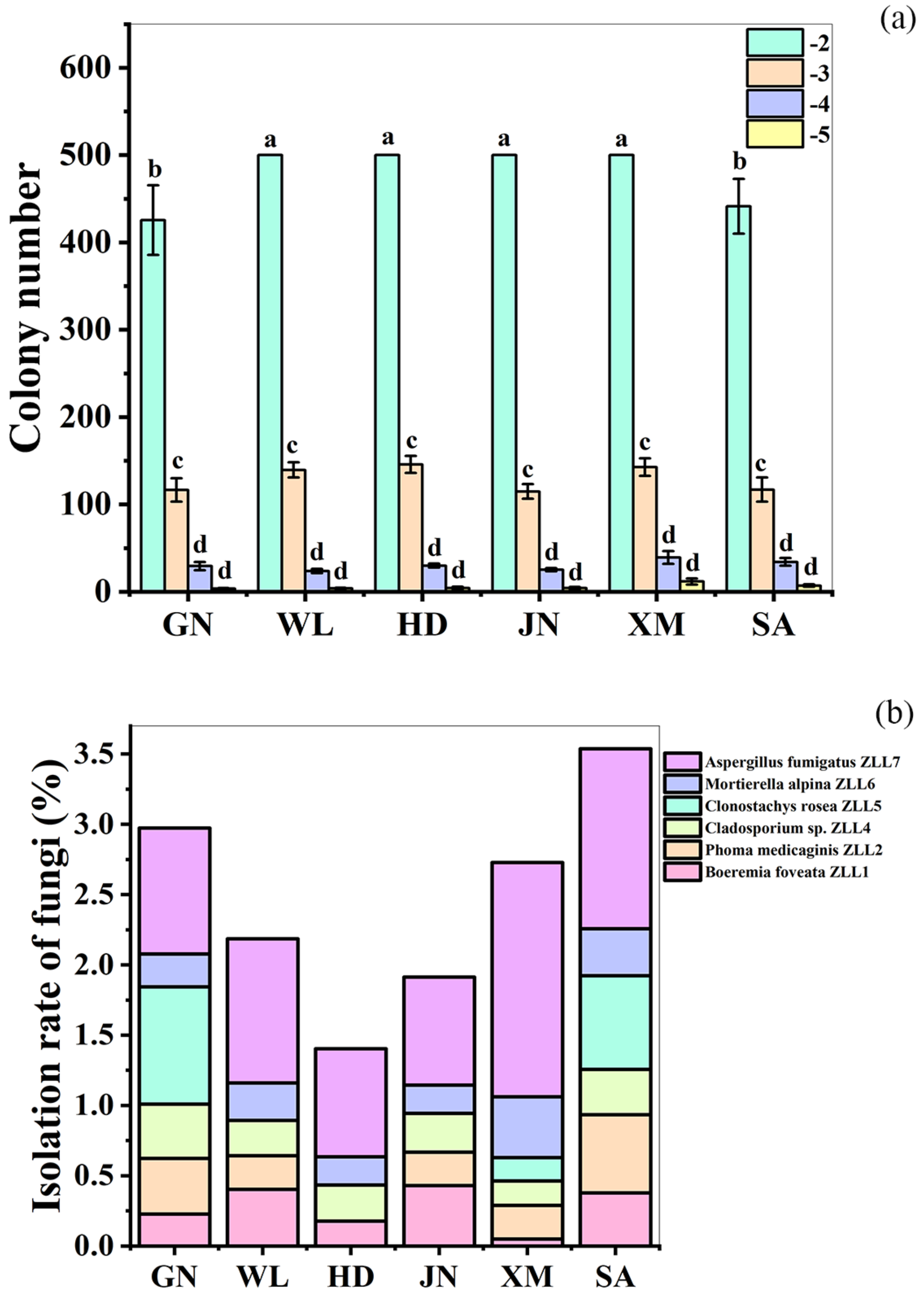

Figure S6. Culturable microorganisms isolated from inter-root soil and plant stalks of different alfalfa varieties. (**a**) Number of culturable bacterial colonies in different concentration gradients of rhizosphere soil; (**b**) isolation rate of culturable fungi in rhizosphere soil. GN: Gannong No.4 alfalfa variety; WL: WL343HQ alfalfa variety; HD: Dryland alfalfa variety; JN: Magnum II; XM: Xinmu No.1 alfalfa variety; SA: Saranac alfalfa variety.

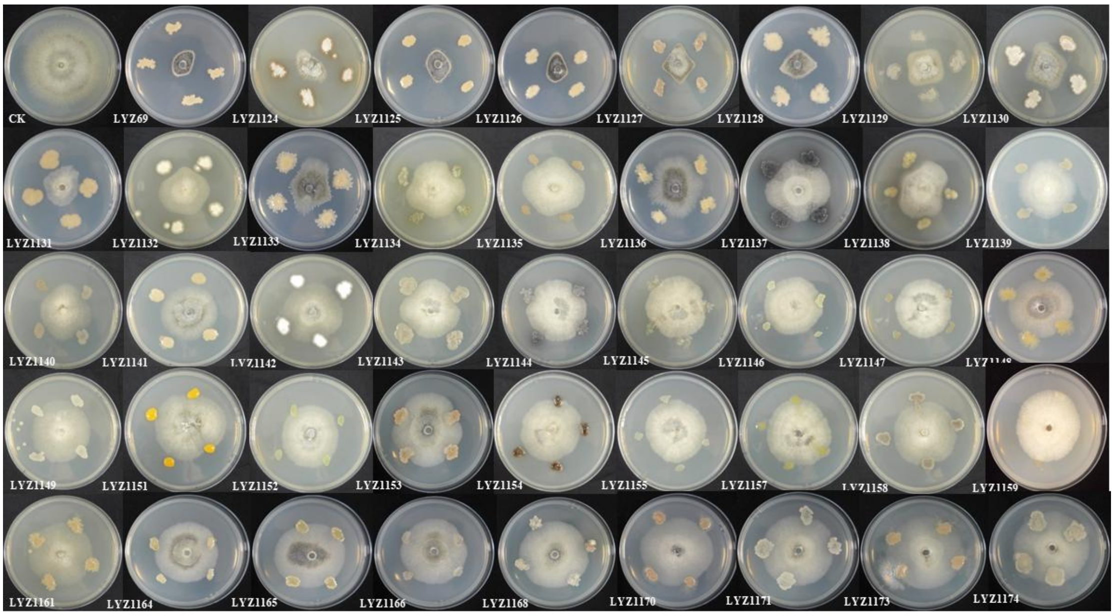

Figure S7. Inhibition zones of *V. alfalfae* LYZ0257 by different bacterial strains.

Table S1 Statistics of rhizosphere soil fungi sequencing data filtration and utilization.

Sample names	SAR number	Bio sample	Region	Reads length (bp)	Raw data (Mbp)	Ploy base (%)	Low quality (%)	Clean data (Mbp)	Data utilization ratio (%)	Raw reads	Clean reads
**GN1**	SRR32342086	SAMN46822339	ITS2	300:300	45	0.107	0.017	42.86	95.25	81607* 2	77447* 2
**GN2**	SRR32342085	SAMN46822340	ITS2	300:300	44.62	0.007	0.044	42.86	96.05	81370* 2	77606* 2
**GN3**	SRR32342074	SAMN46822341	ITS2	300:300	46.75	0.585	0.056	42.86	91.67	85441* 2	77822* 2
**GN4**	SRR32342069	SAMN46822342	ITS2	300:300	45.58	0.272	0.05	42.86	94.02	83570* 2	78049* 2
**WL1**	SRR32342078	SAMN46822355	ITS2	300:300	44.79	0.055	0.03	42.86	95.68	81917* 2	77888* 2
**WL2**	SRR32342077	SAMN46822356	ITS2	300:300	45.08	0.352	0.02	42.86	95.08	82455* 2	78031* 2
**WL3**	SRR32342076	SAMN46822357	ITS2	300:300	44.81	0.074	0.039	42.86	95.64	82453* 2	78327* 2
**WL4**	SRR32342075	SAMN46822358	ITS2	300:300	45.26	0.071	0.028	42.86	94.68	82202* 2	77181* 2
**HD1**	SRR32342068	SAMN46822343	ITS2	300:300	44.71	0.07	0.05	42.86	95.86	82461* 2	78507* 2
**HD3**	SRR32342067	SAMN46822344	ITS2	300:300	44.9	0.172	0.032	42.86	95.45	81544* 2	77455* 2
**HD4**	SRR32342066	SAMN46822345	ITS2	300:300	47.03	1.155	0.027	42.86	91.12	85644* 2	77620* 2
**HD5**	SRR32342065	SAMN46822346	ITS2	300:300	44.82	0.449	0.016	42.86	95.61	81980* 2	77751* 2
**JN1**	SRR32342064	SAMN46822347	ITS2	300:300	47.83	1.77	0.073	42.86	89.6	87723* 2	78007* 2
**JN3**	SRR32342063	SAMN46822348	ITS2	300:300	45.6	1.3	0.017	42.86	93.99	83735* 2	78284* 2
**JN4**	SRR32342084	SAMN46822349	ITS2	300:300	44.91	0.077	0.036	42.86	95.44	82887* 2	78515* 2
**JN5**	SRR32342083	SAMN46822350	ITS2	300:300	45.43	0.567	0.036	42.86	94.34	82639* 2	77472* 2
**XM1**	SRR32342073	SAMN46822359	ITS2	300:300	45.16	0.099	0.025	42.86	94.91	82100* 2	77586* 2
**XM2**	SRR32342072	SAMN46822360	ITS2	300:300	45.1	0.067	0.019	42.86	95.03	82165* 2	77714* 2
**XM3**	SRR32342071	SAMN46822361	ITS2	300:300	44.77	0.25	0.028	42.86	95.73	81989* 2	77871* 2
**XM4**	SRR32342070	SAMN46822362	ITS2	300:300	46.72	0.254	0.072	42.86	91.74	85799* 2	78088* 2

**SA1**	SRR32342082	SAMN46822351	ITS2	300:300	45.15	0.038	0.015	42.86	94.92	83019* 2	78444* 2
**SA3**	SRR32342081	SAMN46822352	ITS2	300:300	44.89	0.089	0.025	42.86	95.47	81828* 2	77582* 2
**SA4**	SRR32342080	SAMN46822353	ITS2	300:300	44.88	0.446	0.026	42.86	95.5	81758* 2	77732* 2
**SA5**	SRR32342079	SAMN46822354	ITS2	300:300	44.54	0.112	0.023	42.86	96.23	81328* 2	77839* 2

Table S2 Statistics of rhizosphere soil bacterial sequencing data filtration and utilization.

**Sample names**	**Run**	**Bio sample**	**Region**	**Reads length (bp)**	**Raw data (Mbp)**	**Ploy base (%)**	**Low quality (%)**	**Clean data (Mbp)**	**Data utilization ratio (%)**	**Raw reads**	**Clean reads**
**GN1**	SRR32342086	SAMN46822339	16S-V3-V4	300:300	43.87	0.009	0.033	42.86	97.7	80348* 2	77888* 2
**GN2**	SRR32342085	SAMN46822340	16S-V3-V4	300:300	43.86	0.006	0.031	42.86	97.72	79421* 2	76843* 2
**GN3**	SRR32342074	SAMN46822341	16S-V3-V4	300:300	43.88	0.018	0.054	42.86	97.68	79377* 2	77037* 2
**GN4**	SRR32342069	SAMN46822342	16S-V3-V4	300:300	43.96	0.012	0.041	42.86	97.5	79825* 2	77312* 2
**WL1**	SRR32342068	SAMN46822343	16S-V3-V4	300:300	43.99	0.018	0.041	42.86	97.43	79703* 2	77152* 2
**WL2**	SRR32342067	SAMN46822344	16S-V3-V4	300:300	43.9	0.017	0.038	42.86	97.62	79738* 2	77288* 2
**WL3**	SRR32342066	SAMN46822345	16S-V3-V4	300:300	43.86	0.021	0.032	42.86	97.7	80087* 2	77451* 2
**WL4**	SRR32342065	SAMN46822346	16S-V3-V4	300:300	44.07	0.026	0.059	42.86	97.25	80402* 2	77650* 2
**HD1**	SRR32342064	SAMN46822347	16S-V3-V4	300:300	43.78	0.016	0.049	42.86	97.88	79909* 2	77565* 2
**HD3**	SRR32342063	SAMN46822348	16S-V3-V4	300:300	44.07	0.01	0.052	42.86	97.24	80808* 2	77860* 2
**HD4**	SRR32342084	SAMN46822349	16S-V3-V4	300:300	43.8	0.016	0.039	42.86	97.85	80398* 2	77950* 2
**HD5**	SRR32342083	SAMN46822350	16S-V3-V4	300:300	43.76	0.016	0.037	42.86	97.94	79225* 2	77010* 2
**JN1**	SRR32342082	SAMN46822351	16S-V3-V4	300:300	43.85	0.027	0.046	42.86	97.74	79516* 2	77165* 2
**JN3**	SRR32342081	SAMN46822352	16S-V3-V4	300:300	43.89	0.019	0.044	42.86	97.66	79992* 2	77559* 2
**JN4**	SRR32342080	SAMN46822353	16S-V3-V4	300:300	43.89	0.019	0.037	42.86	97.65	80150* 2	77722* 2
**JN5**	SRR32342079	SAMN46822354	16S-V3-V4	300:300	44.17	0.028	0.058	42.86	97.03	80645* 2	77945* 2

**XM1**	SRR32342078	SAMN46822355	16S-V3-V4	300:300	44.08	0.032	0.045	42.86	97.23	80923* 2	78001* 2
**XM2**	SRR32342077	SAMN46822356	16S-V3-V4	300:300	43.77	0.015	0.037	42.86	97.92	80385* 2	78153* 2
**XM3**	SRR32342076	SAMN46822357	16S-V3-V4	300:300	43.77	0.03	0.041	42.86	97.91	79327* 2	77188* 2
**XM4**	SRR32342075	SAMN46822358	16S-V3-V4	300:300	43.83	0.019	0.042	42.86	97.77	79550* 2	77344* 2
**SA1**	SRR32342073	SAMN46822359	16S-V3-V4	300:300	43.85	0.024	0.042	42.86	97.73	80000* 2	77699* 2
**SA3**	SRR32342072	SAMN46822360	16S-V3-V4	300:300	43.98	0.023	0.06	42.86	97.45	80478* 2	78103* 2
**SA4**	SRR32342071	SAMN46822361	16S-V3-V4	300:300	44.13	0.017	0.052	42.86	97.11	81077* 2	78212* 2
**SA5**	SRR32342070	SAMN46822362	16S-V3-V4	300:300	44.07	0.015	0.047	42.86	97.25	81071* 2	78334* 2

Table S3 The culturable microorganisms in rhizosphere soil and alfalfa stalks of different alfalfa varieties.

**Number**	**Classification**	**Strains and their designations**	**Separation source**
**1**	Bacteria, Bacillota, Bacilli, Bacillales, Bacillaceae	*Bacillus amyloliquefaciens* LYZ1125	Alfalfa stems
**2**	Bacteria, Bacillota, Bacilli, Bacillales, Bacillaceae	*Bacillus velezensis* LYZ1126	Alfalfa stems
**3**	Bacteria, Bacillota, Bacilli, Bacillales, Bacillaceae	*Bacillus atrophaeus* LYZ1127	Soil
**4**	Bacteria, Bacillota, Bacilli, Bacillales, Bacillaceae	*Bacillus subtilis* LYZ1128	Alfalfa stems
**5**	Bacteria, Bacillota, Bacilli, Bacillales, Bacillaceae	*Bacillus atrophaeus* LYZ1129	Soil
**6**	Bacteria, Bacillota, Bacilli, Bacillales, Bacillaceae	*Priestia aryabhattai* LYZ1131	Alfalfa stems
**7**	Bacteria, Bacillota, Bacilli, Bacillales, Bacillaceae	*Bacillus inaquosorum* LYZ1133	Alfalfa stems

**8**	Bacteria, Bacillota, Bacilli, Bacillales, Paenibacillaceae	*Brevibacillus laterosporus* LYZ1135	Soil
**9**	Bacteria, Bacillota, Bacilli, Bacillales, Bacillaceae	*Bacillus safensis* LYZ1136	Alfalfa stems
**10**	Bacteria, Bacillota, Bacilli, Bacillales, Bacillaceae	*Bacillus pumilus* LYZ1141	Soil
**11**	Bacteria, Bacillota, Bacilli, Bacillales, Bacillaceae	*Peribacillus simplex* LYZ1143	Soil
**12**	Bacteria, Bacillota, Bacilli, Bacillales, Bacillaceae	*Peribacillus simplex* LYZ1144	Soil
**13**	Bacteria, Bacillota, Bacilli, Bacillales, Bacillaceae	*Peribacillus castrilensis* LYZ1145	Soil
**14**	Bacteria, Bacillota, Bacilli, Bacillales, Bacillaceae	*Peribacillus frigoritolerans* LYZ1146	Alfalfa stems
**15**	Bacteria, Bacillota, Bacilli, Bacillales, Bacillaceae	*Bacillus pumilus* LYZ1148	Alfalfa stems
**16**	Bacteria, Bacillota, Bacilli, Bacillales, Bacillaceae	*Peribacillus castrilensis* LYZ1153	Alfalfa stems
**17**	Bacteria, Bacillota, Bacilli, Bacillales, Bacillaceae	*Bacillus idriensis* LYZ1160	Soil
**18**	Bacteria, Bacillota, Bacilli, Bacillales, Bacillaceae	*Bacillus altitudinis* LYZ1161	Soil
**19**	Bacteria, Bacillota, Bacilli, Bacillales, Planococcaceae	*Solibaccillus silvestris* LYZ1159	Alfalfa stems
**20**	Bacteria, Bacillota, Bacilli, Bacillales, Bacillaceae	*Bacillus gibsonii* LYZ1163	Alfalfa stems
**21**	Bacteria, Bacillota, Bacilli, Bacillales, Bacillaceae	*Bacillus australimaris* LYZ1164	Alfalfa stems
**22**	Bacteria, Bacillota, Bacilli, Bacillales, Bacillaceae	*Bacillus altitudinis* LYZ1165	Alfalfa stems
**23**	Bacteria, Bacillota, Bacilli, Bacillales, Bacillaceae	*Bacillus licheniformis* LYZ1166	Alfalfa stems

**24**	Bacteria, Bacillota, Bacilli, Bacillales, Paenibacillaceae	*Paenibacillue harenae* LYZ1167	Alfalfa stems
**25**	Bacteria, Bacillota, Bacilli, Bacillales, Bacillaceae	*Bacillus thuringiensis* LYZ1168	Alfalfa stems
**26**	Bacteria, Bacillota, Bacilli, Bacillales, Bacillaceae	*Bacillus toyonensis* LYZ1170	Alfalfa stems
**27**	Bacteria, Bacillota, Bacilli, Bacillales, Bacillaceae	*Bacillus aerius* LYZ1171	Alfalfa stems
**28**	Bacteria, Bacillota, Bacilli, Bacillales, Bacillaceae	*Bacillus idriensis* LYZ1172	Alfalfa stems
**29**	Bacteria, Bacillota, Bacilli, Bacillales, Bacillaceae	*Bacillus mobilis* LYZ1173	Alfalfa stems
**30**	Bacteria, Bacillota, Bacilli, Bacillales, Bacillaceae	*Bacillus simplex* LYZ1174	Alfalfa stems
**31**	Bacteria, Actinobacteria, Actinomycetes, Streptomycetales, Streptomycetaceae	*Streptomyces galilaeus* LYZ1124	Soil
**32**	Bacteria, Actinobacteria, Actinomycetes, Streptomycetales, Streptomycetaceae	*Streptomyces bobili* LYZ1130	Soil
**33**	Bacteria, Actinobacteria, Actinomycetes, Streptomycetales, Streptomycetaceae	*Streptomyces microflavus* LYZ1132	Soil
**34**	Bacteria, Actinobacteria, Actinomycetes, Streptomycetales, Streptomycetaceae	*Streptomyces anulatus* LYZ1134	Soil
**35**	Bacteria, Actinobacteria, Actinomycetes, Streptomycetales, Streptomycetaceae	*Streptomyces europaeiscabiei* LYZ1138	Soil
**36**	Bacteria, Actinobacteria, Actinomycetes, Micrococcales, Microbacteriaceae	*Microbacterium foliorum* LYZ1137	Soil
**37**	Bacteria, Actinobacteria, Actinomycetes, Streptomycetales, Streptomycetaceae	*Streptomyces umbrinus* LYZ1142	Soil
**38**	Bacteria, Actinobacteria, Actinomycetes, Micrococcales, Micrococcaceae	*Glutamicibacter arilaitensis* LYZ1149	Soil
**39**	Bacteria, Actinobacteria, Actinomycetes, Corynebacteriales, Nocardiaceae	*Rhodococcus sovatensis* LYZ1151	Soil
**40**	Bacteria, Actinobacteria, Actinomycetes, Micrococcales, Microbacteriaceae	*Leucobacter chromiiresistens* LYZ1152	Soil
**41**	Bacteria, Actinobacteria, Actinomycetes, Micrococcales, Micrococcaceae	*Arthrobacter bambusae* LYZ1154	Soil
**42**	Bacteria, Actinobacteria, Actinomycetes, Micrococcales, Micrococcaceae	*Arthrobacter ruber* LYZ1156	Soil

**43**	Bacteria, Actinobacteria, Actinomycetes, Micrococcales, Microbacteriaceae	*Microbacterium phyllosphaerae* LYZ1155	Soil
**44**	Bacteria, Proteobacteria, Gammaproteobacteria, Pseudomonadales, Pseudomonadaceae	*Pseudomonas baetica* LYZ1139	Soil
**45**	Bacteria, Proteobacteria, Gammaproteobacteria, Pseudomonadales, Pseudomonadaceae	*Pseudomonas arenae* LYZ1140	Soil
**46**	Bacteria, Proteobacteria, Gammaproteobacteria, Pseudomonadales, Pseudomonadaceae	*Pseudoxanthomonas mexicana* LYZ1147	Soil
**47**	Bacteria, Proteobacteria, Gammaproteobacteria, Pseudomonadales, Pseudomonadaceae	*Pseudomonas migulae* LYZ1150	Soil
**48**	Bacteria, Actinobacteria, Actinomycetes, Micrococcales, Micrococcaceae	*Schumannella luteola* LYZ1157	Soil
**49**	Bacteria, Proteobacteria, Gammaproteobacteria, Xanthomonadales, Xanthomonadaceae	*Stenotrophomonas maltophilia* LYZ1158	Soil
**50**	Bacteria, Bacteroidetes, Sphingobacteriia, Sphingobacteriales, Sphingobacteriaceae	*Pedobacter steynii* LYZ1162	Soil
**51**	Fungi, Ascomycota, Dothideomycetes, Pleosporales, Didymellaceae	*Boeremia foveata* ZLL1	Soil
**52**	Fungi, Ascomycota, Dothideomycetes, Pleosporales, Didymellaceae	*Phoma medicaginis var. medicaginis* ZLL2	Soil
**53**	Fungi, Ascomycota, Dothideomycetes, Capnodiales, Cladosporiaceae	*Cladosporium* sp. ZLL4	Soil
**54**	Fungi, Ascomycota, Sordariomycetes, Hypocreales, Bionectriaceae	*Clonostachys rosea* ZLL5	Soil
**55**	Fungi, Mucoromycota, Mucoromycetes, Mucorales, Mortierellaceae, Mortierella	*Mortierella alpina* ZLL6	Soil
**56**	Fungi, Ascomycota, Eurotiomycetes, Eurotiales, Aspergillaceae	*Aspergillus fumigatus* ZLL7	Soil
