# Supplementary figures and images for: Effect of alfalfa varieties with different resistance to alfalfa Verticillium wilt on microbial communities in rhizosphere soil and plants
Source: Front Microbiol. 2026 May 19;17:1739219. doi: 10.3389/fmicb.2026.1739219 (PMC13226603; doi:10.3389/fmicb.2026.1739219)

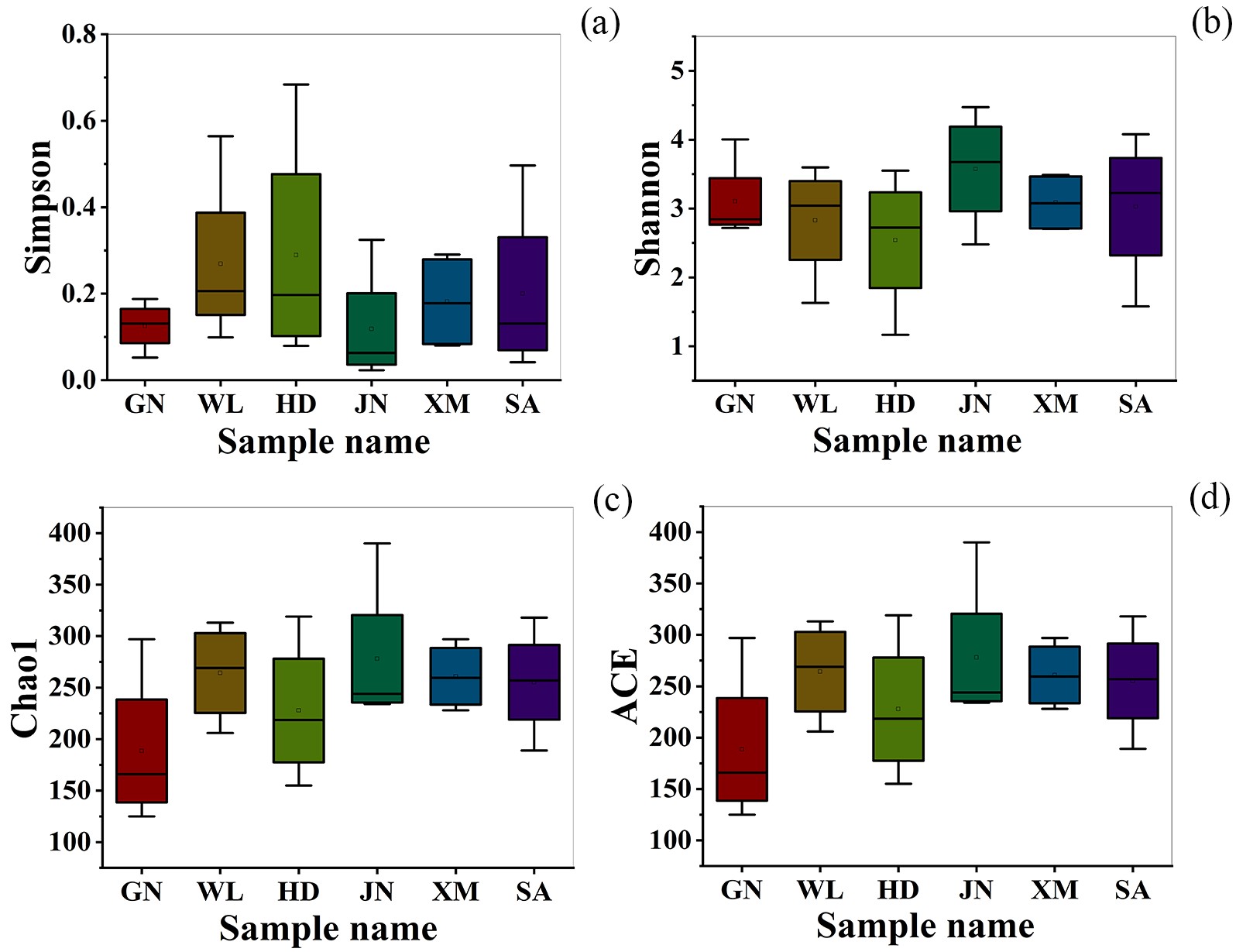

Supplement: Supplementary file 2 [file Image_2.jpeg]
